# Histidine in Proteins: pH-Dependent Interplay between π–π, Cation–π, and CH–π Interactions

**DOI:** 10.1021/acs.jctc.4c00606

**Published:** 2024-07-22

**Authors:** Rivka Calinsky, Yaakov Levy

**Affiliations:** Department of Chemical and Structural Biology, Weizmann Institute of Science, Rehovot 76100, Israel

## Abstract

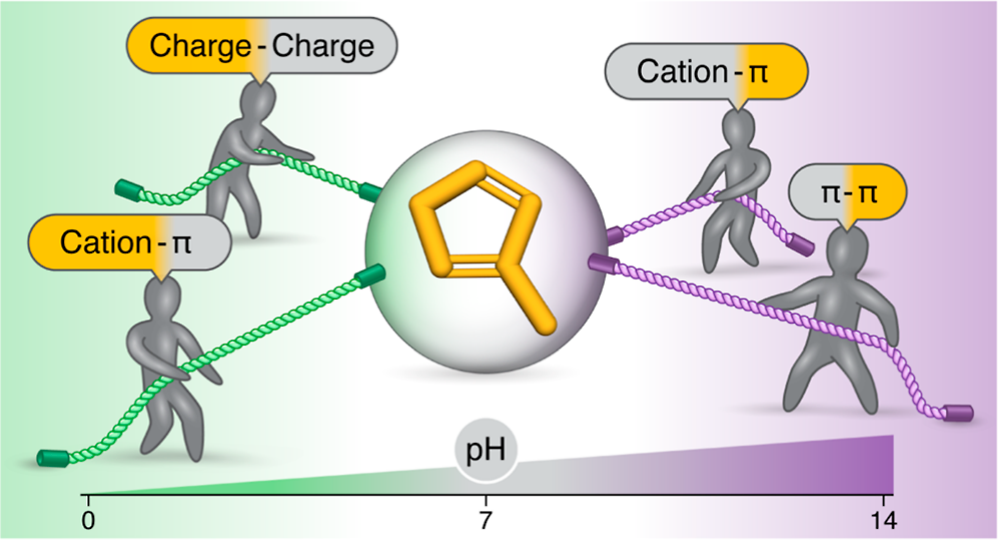

Histidine (His) stands out as the most versatile natural amino acid due to its side chain’s facile propensity to protonate at physiological pH, leading to a transition from aromatic to cationic characteristics and thereby enabling diverse biomolecular interactions. In this study, our objective was to quantify the energetics and geometries of pairwise interactions involving His at varying pH levels. Through quantum chemical calculations, we discovered that His exhibits robust participation in both π–π and cation–π interactions, underscoring its ability to adopt a π or cationic nature, akin to other common residues. Of particular note, we found that the affinity of protonated His for aromatic residues (via cation–π interactions) is greater than the affinity of neutral His for either cationic residues (also via cation–π interactions) or aromatic residues (via π–π interactions). Furthermore, His frequently engages in CH–π interactions, and notably, depending on its protonation state, we found that some instances of hydrogen bonding by His exhibit greater stability than is typical for interamino acid hydrogen bonds. The strength of the pH-dependent pairwise energies of His with aromatic residues is supported by the abundance of pairwise interactions with His of low and high predicted p*K*_a_ values. Overall, our findings illustrate the contribution of His interactions to protein stability and its potential involvement in conformational changes despite its relatively low abundance in proteins.

## Introduction

Histidine (His) is one of the nine essential amino acids that cannot be sufficiently synthesized by some organisms and is relatively rare, with a frequency of a mere 2.3% in proteins. Remarkably, histidine is found nevertheless in approximately 50% of all catalytic sites, indicating its crucial role in biological systems.^1^ In addition, some proteins are rich with His residues, which are either spread along the protein sequences or clustered as long stretches of His (i.e., repeat sequences).^2^ Among the 20 natural amino acids, His can be considered the most versatile in terms of protein structure and function. The versatility of His is attributed to its imidazole side chain, which has aromatic characteristics. Unique among its aromatic counterparts (Phe, Tyr, and Trp), the His imidazole side chain exhibits an acidic ionization constant (p*K*_a_) of 6.3,^3^ which is near the physiological pH. This characteristic p*K*_a_ facilitates the ability of His to switch between neutral and positively charged states in response to small variations in pH. This transition capability plays a pivotal role in biological phenomena involving structural changes, such as the gating of the proton channel in the influenza virus.^4^ Furthermore, the reported acidic pH in the brain of patients with Alzheimer’s disease (6.6 on average compared to 7.0 in healthy brain^5^) has been recently linked to the aggregation of β-amyloid peptide, where the increase of protonated His portion^6^ promotes increased β-sheet content formation and thus misfolding of the peptide.^7,8^

Often, the pH-dependent protonation state of His is linked to a switch in the nature of its molecular interactions. The pH was found to strongly affect the nature of His partners (hydrophobic vs hydrophilic) and thus preferred interactions of His.^9^ Most commonly, at low pH (pH < p*K*_a_ – 1), where His is likely to be protonated and therefore positively charged, it can participate in electrostatic interactions with charged residues (Glu, Asp, Lys, and Arg).^10^ At a higher pH (pH > p*K*_a_ + 1), where His is neutral, it can participate in aromatic π–π interactions. However, the rich literature discussing aromatic interactions frequently features Phe, Tyr, and Trp,^11−17^ whereas the inclusion of His in these studies remains somewhat sparse,^18−24^ which may be attributable to its multiple protonation states adding layers of complexity to the analyses.^23,25^

Beyond its participation in aromatic π–π interactions, His can also participate in cation–π interactions. Cation–π interactions involving His are particularly unique because, depending on the pH, His can participate either as a cation (electron acceptor) or as a π system (electron donor). At low pH, where His is positively charged, His serves as a cation and may interact with π systems (i.e., Phe, Tyr, Trp, or another His residue in the neutral state). At high pH, where His is uncharged, His serves as a π–system and may interact with charged residues (i.e., Lys and Arg).^23^ At pH ≈ p*K*_a_, where some of the histidine residues are charged and others are neutral, cation–π interactions can be formed between two His residues in different protonation states (i.e., between an imidazole group and an imidazolium ion). In addition to the participation of His in π–π, cation–π, and charge–charge interactions, it can be involved in other types of interactions. In the neutral state, His is a powerful metal ion coordinator. The basic nitrogen atom of the imidazole group is found in many cases to coordinate metallic cations such as Ca^2+^, Zn^2+^, Ni^2+^, or Cu^2+^. Furthermore, His can engage in hydrogen bonding because the polar hydrogen or the basic nitrogen of its imidazole group may serve as a hydrogen bond (H-bond) donor or acceptor, respectively.^26^ Finally, CH–π interactions involving His, although rarely highlighted in the literature,^27^ are remarkably common, particularly given its relatively low natural abundance.^28,29^

Quantifying the interaction geometry and energetics of histidine residues within proteins demands knowledge of their protonation state. However, distinguishing between neutral and charged His states^23^ is challenging due to the limitations on directly observing hydrogen atoms in X-ray structures.^30^ As such, insights into the geometric conformations of His interactions cannot be clearly separated and attributed to either the protonated or neutral state.^28^ Investigations primarily centered around energy aspects tend to either represent His through the neutral imidazole^31^ or focus exclusively on its charged state.^32^ This challenge extends to identifying the protonation state in existing Protein Data Bank (PDB) structures. Although neutron diffraction methods^30^ offer potential solutions, the scarcity of nonredundant neutron diffraction structures in PDB data sets limits their widespread application. Various computational approaches aim to bridge these gaps by predicting the protonation states of histidine in proteins at a given pH; yet, their degree of accuracy is limited.^3,33−41^

The current study aims to quantify the range of molecular interactions engaged in by neutral and protonated His residues in proteins by means of a comprehensive energetic analysis using quantum calculations of the interactions of His with several amino acids (Phe, Tyr, Trp, Lys, and Arg). Our study allows quantifications of the energetic strengths of the π–π, cation–π, CH–π, and hydrogen bonding interactions engaged in by His in various protonation states and geometries. Furthermore, our analyses enable a comparison of the characteristics of π–π and cation–π interactions involving His with those involving other amino acids of a similar nature that are often found in proteins.

## Methods

Our analysis utilized two unique sets of PDB structures to explore interacting histidine-containing residue pairs. A primary data set was assembled from PISCES,^42^ drawing on 6535 high-resolution (resolution ≤ 1.8 Å and *R*-factor ≤ 0.18) X-ray structures screened for nonredundancy and length (40–10,000 residues), as outlined in a previously published study.^43^ Additionally, we supplemented this with a set of 74 structures (*R* ≤ 2.5 Å) determined through neutron diffraction, which are particularly valuable, as they show the positions of protons on histidine residues. While not all protons are visible even with such a technique, we found the use of deuterated determined structures to be a trustworthy indication for His protonation state.^44^

**Figure 1:**
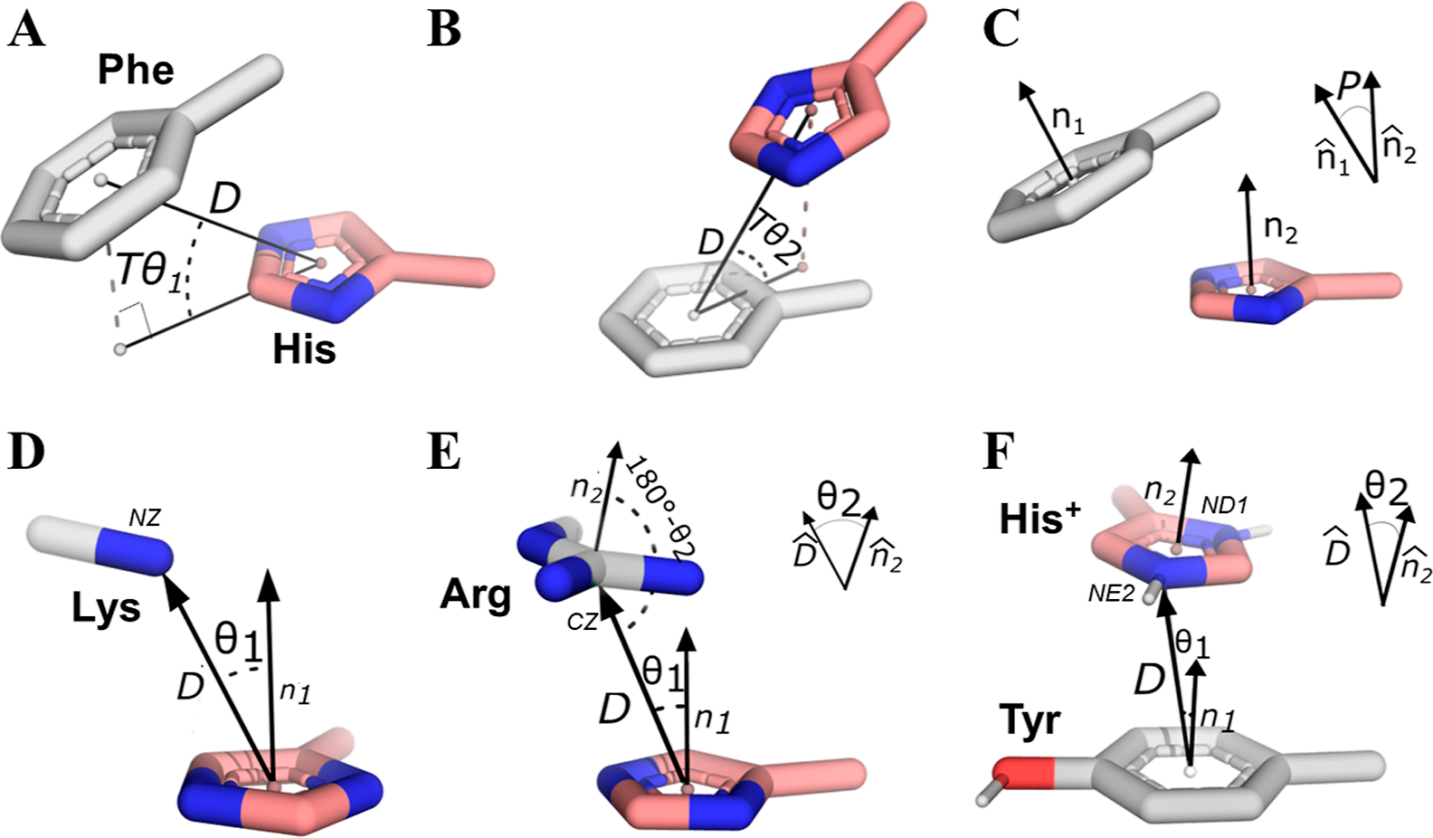
Selected geometrical parameters to represent aromatic–aromatic and cationic–aromatic interaction conformations involving histidine. (A–C) Definition of parameters for a His–Phe pair, where *D* is the distance between the centroids of the His ring (pink with the nitrogen atoms colored in blue) and the Phe ring (gray), Tθ_1_ is the elevation of the Phe centroid relative to His, Tθ_2_ is the elevation of His relative to the Phe centroid, and *P* denotes the angle between the normal vectors of the aromatic rings of the paired His and Phe residues, provided *P* ≤ 90°. (D–F) Definition of parameters for amino acid pairs involving His and Lys, Arg, or Tyr based on ref (24). (D) Parameters for a His^0^–Lys pair, where θ_1_ describes the angle between the NZ atom of Lys and the normal of the His ring’s plane (represented by the n1 vector), where *D* is the distance of the NZ atom from the ring centroid. (E) For the His^0^–Arg pair, an additional parameter, θ_2_, denotes the angle of the normal to the Arg’s plane and the direction of the vector *D*. (F) Parameters for a His^+^–Tyr pair, where the distances and angles are taken from the closest His nitrogen (NE2 or ND1) for a given pair. The geometric parameters (panels A–C) are illustrated for His–Phe, with the same parameters used for the other pairwise interactions (as His–Trp). All the pairwise interactions between two rings (i.e., aromatic–aromatic pairs and cationic–aromatic pairs in which His serves as the cation) were characterized using the three parameters *D*, *P*, and Tθ_2_, being the elevation of His from its partner’s ring. We note that, in the definition of Tθ_1_ in the context of homogeneous His–His pairs, this angle refers only to the order of their assignment (with His chosen first as the reference). In these cases, both elevation angles were calculated for the two scenarios. All cationic–aromatic systems (panels D–F) were characterized using *D* and θ_1_. For His^+^ and Arg as cations, also θ_2_ was considered.

Prior to geometric and energetic analysis, we focused on quantifying the relative occurrences of distinct His tautomers in the neutron diffraction data set, limiting the analysis to deuterated cases only. These tautomers are labeled His_ε_^0^, His_δ_^0^, and His^+^ according to the position of their protonated nitrogen,^45^ namely, NE2, ND1, or both, respectively (see Figures 1 and S1). Upon analysis, we discovered 122 instances of His_ε_^0^, 64 instances of His_δ_^0^, and 94 instances of His^+^. Observing the greater prevalence of His_ε_^0^ over His_δ_^0^ (which is in line with conclusions of experimental work^46^), we selected His_ε_^0^ for comparison with His^+^ in this work. For simplicity, we will refer to His_ε_^0^ as His^0^.

### Aromatic–Aromatic Pair Parameters

We started our investigation by identifying pairs of interacting aromatic amino acids. We applied a carbon–carbon approach previously defined for Phe–Phe pairs^47^ and broadened it to include other aromatic amino acids: Tyr, Trp, and His. To focus the analysis on relevant interactions, sequences of five or more consecutive His residues were considered His-tags and excluded, as was any His residue located within 5 Å of a metal cation.

For each pairwise conformation, we calculated two independent variables: the distance between the centroids (*D*) and the angle between aromatic ring planes (*P*). We further considered projected angles Tθ_1_ and Tθ_2_, being the elevation of the centroid of the Phe ring from the plane of the His ring, and the elevation of His ring centroid from the plane of the Phe ring, respectively.^47^ These parameters are further described in Figure 1A–C.

Using these metrics, we sought to pinpoint specific geometries to represent the occupied conformational spaces of selected residue pairs. Therefore, we conducted data clustering by applying the Gaussian mixture model, which clusters spatial data, in our case, the PDB pairs, with an emphasis on the variance in the data. Thus, this allows the clusters to adapt more abstract shapes (i.e., not limited to spherical shapes) than more commonly used methods like K-Means. The implementation of the clustering algorithm was done using the scikit-learn library.^48^ For the smaller neutron diffraction data set, we considered all defined pairs without clustering to ensure no important conformations were excluded.

Binding energies (being the difference in energy between the optimized pairwise conformation and the energies of the optimized separated residues) for the representative pairs, selected based on clustering the sampled pairs, were then calculated using the ORCA 5.0.3^49^ software. For this purpose, each pair geometry was first briefly (<500 steps) optimized using the double-hybrid functional revDSD-PBE86-D4/QZ^50^ with the Conductor-like Polarizable Continuum Model (CPCM) as an implicit water solvent. The use of polarizable implicit model within dispersion-corrected density functional theory (DFT-D) calculations was previously shown to describe intermolecular interactions of biologically relevant molecules in water, as accurate in the gas phase.^51^ The CPCM model was previously used for calculations of p*K*_a_ of protonated and neutral (solvent exposed) His residues, providing a great balance in computational time and accuracy.^52^ The optimized geometries indicate a proximate local or global minimum energy conformation. Although our initial clustered points sampled the geometric space within the PDB, the optimized conformations might converge toward a distinct geometry. This convergence may indicate the influence of the protein’s environment on the isolated pairwise interactions.

The quantum mechanics (QM) parameters were chosen based on their high performance for ion–π data sets and π-stacking interaction data sets.^53,54^ To account for the polarity of His, the reported binding energies were calculated using diffused basis set def2-qzvppd’ (excluding hydrogen atoms) for revDSD-PBE86-D4, after a short validation for 19 pairs at the LNO-CCSD(T)^55^ level of calculations. For the latter, we applied the aug-cc-pV5Z basis set (cc-pV5Z for H atoms) using the MRCC program,^56^ as shown in Supporting Information Table S1 and Figure S2. These validations suggest that the binding energies on an average deviate by 0.2 kcal/mol (∼1.8% error of value) from the higher level calculations. To further increase the performance, the representative structures included only one carbon that was additional to the ring, namely, 4-methylimidazole for neutral His and toluene for Phe, as demonstrated in Figures S1 and S2.

The energies of His pairs were calculated separately for neutral His (protonated at the NE2, namely, the Nε2 position^57^) and for positively charged His^+^. The binding energies of the other pairs were calculated to serve as a reference (Table 1).

**Table 1:** Average Binding Energies (kcal/mol) of Pairwise Aromatic–Aromatic Interactions Involving His^a^

	CH–π^b^		stacked^b^		H-bonds^b^	
	solvent	gas phase	solvent	gas phase	solvent	gas phase
His^0^–His^0^	–2.4 ± 0.3	–2.7 ± 0.8	–3.0 ± 0.2	–3.3 ± 1.1	–5.7 ± 0.9	–9.4 ± 1.4
			(–3.9 ± 0.5)^c^	(–8.3 ± 1.9)^c^		
			(–2.6 ± 0.4)^d^			
His^0^–Phe	–2.7 ± 0.3	–3.4 ± 0.6	–3.1 ± 0.4	–3.5 ± 0.9		
			(–4.1 ± 0.5)^e^	(–9.6 ± 1.2)^e^		
His^0^–Tyr	–3.0 ± 0.3	–3.7 ± 0.8	–3.2 ± 0.3	–3.9 ± 0.8	–6.4 ± 2.4	–8.9 ± 2.9
			(–4.5 ± 0.5)^e^	(–10 ± 1.4)^e^		
His^0^–Trp	–3.2 ± 0.8	–3.9 ± 1.5	–4.0 ± 0.4	–4.9 ± 1.0	–5.4 ± 0.8	–8.8 ± 1.5
			(–5.6 ± 0.7)^e^	(–14 ± 2.4)^e^		
Phe–Phe	–2.9 ± 0.3	–3.4 ± 0.4	–3.3 ± 0.3	–3.7 ± 0.5		
Phe–Tyr	–3.1 ± 0.3	–3.7 ± 0.5	–3.5 ± 0.3	–4.0 ± 0.7		
Phe–Trp	–3.4 ± 0.6	–4.2 ± 0.9	–4.2 ± 0.5	–4.7 ± 0.5		
Tyr–Tyr	–3.4 ± 0.4	–4.1 ± 0.8	–3.7 ± 0.3	–4.3 ± 0.6	–4.8 ± 0.3	–6.5 ± 0.4
Tyr–Trp	–3.7 ± 0.6	–4.5 ± 1.1	–4.4 ± 0.6	–5.0 ± 0.8	–4.4 ± 0.5	–6.6 ± 0.4
Trp–Trp	–4.2 ± 0.6	–5.0 ± 1.1	–5.2 ± 0.43	–5.3 ± 0.7		

a Only interactions whose binding energy falls below the −1 kcal/mol threshold are considered.

b CH–π, stacked, and H-bonding pairwise interactions were classified based on their geometrical parameters, as described in Methods.

c Values refer to the interaction between His^0^ and His^+^.

d Values refer to the interaction between His^+^ and His^+^.

e Values refer to the interaction between His^+^ and the corresponding aromatic residue.

### Parameters for Cationic–Aromatic Pairs

We extended our analysis to pairs involving aromatic and positively charged amino acids (cationic–aromatic pairs) using the geometric parameters obtained from another work,^24^ as demonstrated in Figure 1D,E. To first include His as a cation, we extended the previously published definition to account for any of the doubly protonated His nitrogen atoms, as shown in Figure 1F.

These pairs were similarly clustered based on their geometric characteristics, providing 72 representative pairs for the larger data set, along with all defined pairs for the smaller neutron diffraction data set.

### Classification of His Pairwise Interactions

To compare the abundance and strengths of typically discussed interactions, we categorized hydrogen-bonded pairs,^58^ CH–π pairs,^27^ π-stacking interactions,^59^ and cation–π^60^ pairs, as defined by geometric criteria established in previous research. These definitions rely on precise data concerning the hydrogen atom positions, and consequently, we utilized only the QM-optimized pairwise configurations for these categorizations. We consider only geometries satisfying binding energies lower than −1 kcal/mol for any categorization of interactions (H-bond, CH–π, etc.), which is at least 5-folds greater than the average error for these calculations (see Supporting Information Table S1). We further note that the standard deviations for the average energies reported for each interaction (see Tables 1 and 2) might be overestimated as it includes geometries that span different conformational angular regions of our parameters space. For the estimation of error coming from using fixed geometries, refer to Supporting Information Section S3.

**Table 2:** Average Binding Energies (kcal/mol) of Pairwise Cationic–Aromatic Interactions Involving His^a^

	CH–π^b^		cation–π^b^		H-bonds^b^	
	solvent	gas phase	solvent	gas phase	solvent	gas phase
His^0^–Lys	–1.9 ± 0.1	–8.0 ± 1.5	–2.0 ± 0.4	–9.4 ± 2.6	–6.7 ± 2.6	–28 ± 8.8
His^0^–Arg	–3.3 ± 0.1	–7.8 ± 1.9	–3.4 ± 0.3	–8.2 ± 1.2	–6.3 ± 1.4	–22 ± 6.6
His^0^–His^+^	–2.4 ± 0.6	–7.2 ± 2.8	–3.7 ± 0.6	–8.5 ± 1.9	–8.8 ± 1.3	–26 ± 4.0
His^+^–Phe	–2.8 ± 0.6	–7.0 ± 3.3	–3.9 ± 0.6	–10 ± 1.4		
His^+^–Tyr	–3.4 ± 0.9	–9.6 ± 3.5	–4.3 ± 0.6	–11 ± 1.7	–4.8 ± 0.2	–15 ± 0.6
His^+^–Trp	–3.7 ± 1.3	–11 ± 4.2	–5.4 ± 1.0	–15 ± 2.0		
Phe–Lys	–2.2 ± 0.2	–12 ± 1.6	–2.4 ± 0.4	–12 ± 3.5		
Tyr–Lys	–2.3 ± 0.2	–12 ± 1.6	–2.3 ± 0.4	–12 ± 2.7	–3.9 ± 0.5	–18 ± 0.6
Trp–Lys	–3.0 ± 0.7	–15 ± 3.7	–3.1 ± 0.8	–16 ± 4.9		
Phe–Arg	–3.2 ± 0.4	–8.8 ± 0.8	–3.4 ± 0.4	–10 ± 1.5		
Tyr–Arg	–3.4 ± 0.4	–9.6 ± 1.2	–3.7 ± 0.4	–11 ± 1.3	–4.0 ± 0.3	–13 ± 1.5
Trp–Arg	–4.4 ± 0.6	–12 ± 1.2	–4.7 ± 0.6	–15 ± 2.3		

a Only interactions whose binding energy falls below the −1 kcal/mol threshold are considered.

b CH–π, cation–π, and H-bonding of pairwise interactions were classified based on their geometrical parameters, as described in Methods.

Π-Stacking interactions were identified based on the distance cutoff between the centroids of the aromatic rings and using angle definitions that define the orientation of the planes of the residues as approximately parallel. To characterize the unique cation–π interactions in which His serves as the cation (Figure 1F), we extended the definition of Lys and Arg cations (Figure 1D,E) to consider the distance from the heavy, positively charged center atom to the center of the ring. Since both nitrogen atoms are protonated, we chose the one closest to the center of the π system for distance cutoff calculations. CH–π interactions are defined by the distance of the closest carbon (which donates hydrogen) to the center of the π-acceptor system along with the projected distance of the donated hydrogen on the π-system from this center. Finally, the identification of H-bonds includes determining the distance between the hydrogen donor and acceptor; the angle between the donor, the hydrogen atom, and the acceptor atom; and the angle of H-bond from the His’ ring plane. Additional details of these geometric classifications can be found in Supporting Information Figure S4.

### p*K*_a_ Calculations of His in the High-Resolution PDB Structures

p*K*_a_ calculations were performed using the PypKA Poisson–Boltzmann based tool,^35^ for all the His in the high-resolution X-ray data set (total 36,393 p*K*_a_ values), where His residues interacting with metals were excluded from this analysis. This tool was chosen based on its performance and relatively low error.^35^ All the His were categorized into three groups: “Low pKa” His as those with p*K*_a_< 5.3, “High pKa” His for those that satisfy p*K*_a_> 7.3, and “Medium pKa” for His with 5.3 ≤ p*K*_a_ ≤ 7.3.

## Results and Discussion

To evaluate the effect of the His protonation state on its pairwise interactions, we separately studied His participation in π–π (Figure 1A–C) and cation–π (Figure 1D–F) pairwise interactions. We selected residue pairs involved in a range of scenarios associated with protein structural and functional dynamics. The participation of His in π–π interactions is studied in His^0^–X pairs, where X = Phe, Tyr, Trp, or His^0^. The participation of His in cation–π interactions is studied when His is in either the deprotonated state (as the π donor) or the protonated state (as the π-accepting cation). The former scenario is investigated using His^0^–X pairs, where X = Lys, Arg, or His^+^, assuming that both Lys and Arg are in their protonated forms, whereas the latter scenario is investigated using His^+^–X pairs, where X is an aromatic residue.

To evaluate the energetics and geometries of the pairwise interactions of His with aromatic or basic amino acids, various configurations of these pairs were sampled from high-resolution protein structures collected from the PDB. Since the protonation state of His in protein structures is often unknown, the sampled pairwise interactions of His with aromatic residues (i.e., Phe, Tyr, Trp, or His) were used to study both π–π and cation–π interactions by assuming the His^0^ or His^+^ states, respectively.

For both types of interactions, we compared the QM-derived interactions of pairs in which His participates with conventional π–π interactions between other aromatic residues (pairwise interactions between Phe, Tyr, and Trp) and with conventional cation–π interactions between non-His residues (pairwise interactions between Phe and Tyr or Trp and between Lys or Arg), whose energetics were reported previously^61^ and that are used here as a reference for the corresponding interaction between His and the relevant residues.

We included binding energy calculations performed under both solvent and gas-phase conditions to compare the contribution of solvent exposure with buried pairwise interactions and to enable comparison with previously reported values, which were often obtained under gas-phase conditions.^31,62^ Given the polarity of His and its charged state, our discussion focuses primarily on solvated conditions.

### His–Aromatic Interactions Can Be Stabilized by Stacking Conformations across Varied pH Values

Our initial objective was to discern the geometries and energetics of interactions between His and aromatic residues (i.e., Phe, Tyr, Trp, and His). For this purpose, the QM binding energies of these His-aromatic interactions were compared with the energetics of other pairs of aromatic residues (Table 1), which were categorized, on the basis of the geometric parameters of the interaction, as π–π or “stacked”, CH–π, or H-bonding (Figure S4). In discussing these interactions, we focus first on the interactions of His–Phe pairs because Phe has a small π system and is incapable of participating in hydrogen bonding.

**Figure 2:**
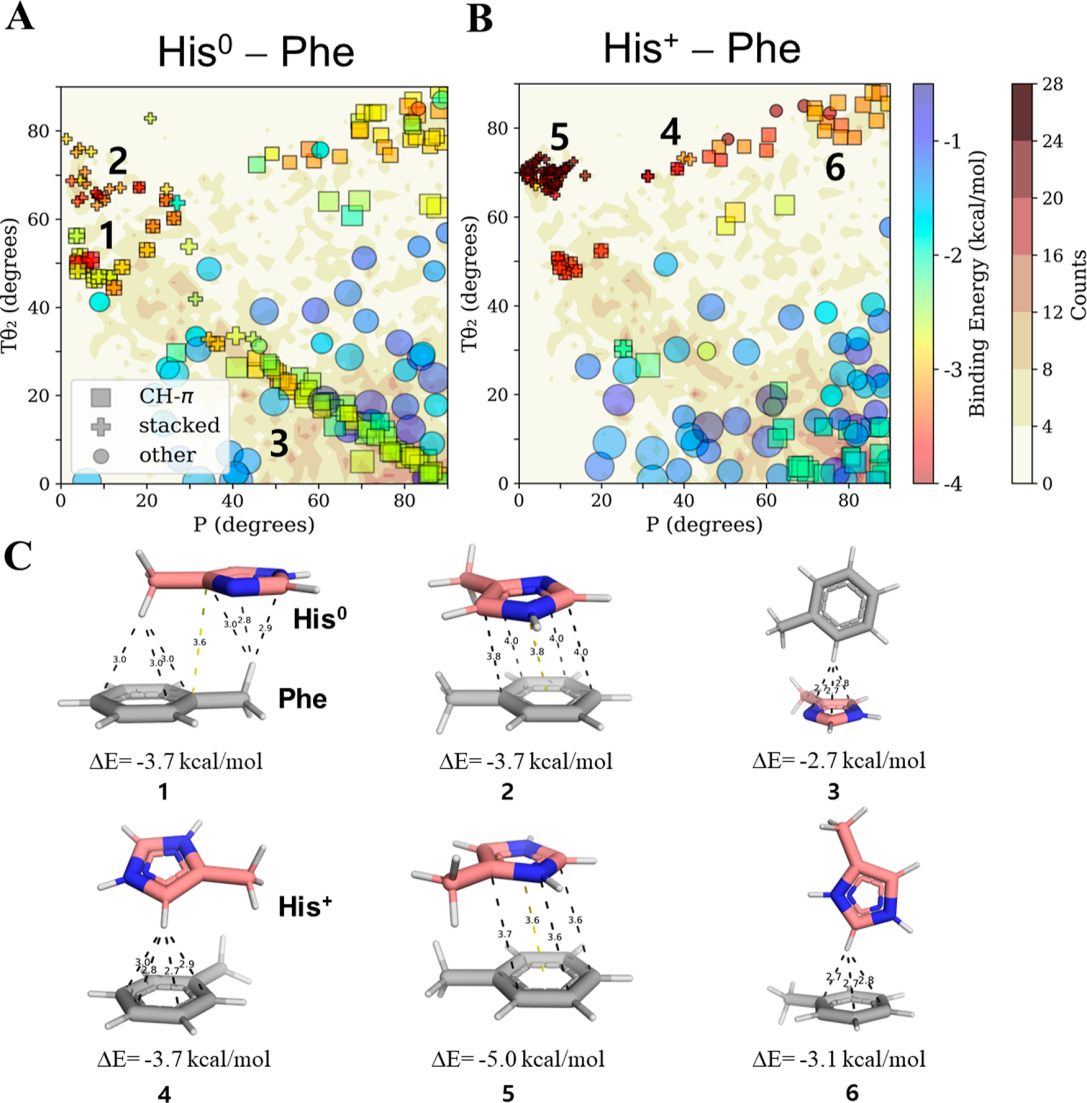
π–π and cation–π interactions between His and Phe at different pH values. Binding energies (rainbow color bar) from QM calculations of His–Phe pairs in solution for pairwise interactions between (A) Phe and His^0^ (at high pH) and (B) Phe and His^+^ (at low pH) projected onto density contour gradients (white–brown color bar) created by counting the frequency of each His–Phe pair geometry as sampled from 6609 high-resolution PDB structures. The geometries of His–Phe are mapped in terms of angular parameters P and Tθ_2_, these being two of the six geometric measures required to represent all pairwise interactions (see Figure 1). The density counts include all PDB pairs from both neutron diffraction and X-ray data sets. The quantum calculations were performed on selected pairs from those found in the sampled database. We note that each selected pair underwent energetic optimization and consequently its final geometry may deviate from its original starting structure. The pairwise interactions are categorized as stacked or CH–π based on distance and angle cutoffs (see Methods and Figure S4 for further details). All other geometries are classified as “other”. The size of the symbol represents the *D* geometric parameter (i.e., the distance, *D*, between the centroids of the Phe and His rings). The stacked geometries for His^0^–Phe and His^+^–Phe may refer to π–π and cation–π, respectively. The numbers overlaid onto the maps correspond to the geometries depicted in panel C. (C) Selected pairwise geometries and their corresponding binding energies for His^0^–Phe pairwise interactions (geometries **1**–**3**; see also panel A) and His^+^–Phe pairwise interactions (geometries 4–6, see also panel B). Pairwise interactions 1 and 2 are minimal energy geometries His^0^–Phe characterized by CH–π or π–π interactions, respectively. Geometry 3 shows a pairwise His^0^–Phe interaction involving a perpendicular CH–π interaction in which His serves as the hydrogen acceptor. Geometries 4 and 5 show minimal energy conformations for CH–π and cation–π interactions, respectively, in His^+^–Phe pairs. Geometry 6 shows a His^+^–Phe pair engaged in a perpendicular CH–π interaction (with His serving as the hydrogen donor).

The interaction energies of His^0^–Phe and His^+^–Phe pairs of different geometries that were sampled from resolved protein structures are shown in Figure 2A,B, respectively. Of the six geometric criteria required to represent all the pairwise interactions between the two ring systems, two (namely, angles Tθ_2_ and *P*, see Figure 1), are plotted in Figure 2. The resulting maps show that His–Phe pairs in proteins are found in various geometries with different stabilities. The most stable His–Phe interactions are those classified as stacked (i.e., π–π) or CH–π, regardless of the His protonation state. For example, Figure 2C highlights stacked geometries possessing low binding energies for His^0^–Phe (geometries 1 and 2) and His^+^–Phe (geometry 5). We note that some geometries meet the classification criteria for both CH–π and stacked interactions, indicating that both interaction types contribute to the same pairwise geometry (e.g., geometry 1). Interactions of the CH–π type are discussed in greater detail later.

The mean binding energies of aromatic–aromatic stacking interactions involving His^0^–Phe and His^+^–Phe are about −3.1 and −4.1 kcal/mol, respectively (see Table 1). This energy difference may be attributed to the different nature of the stacking interactions engaged in by His^0^–Phe compared with that of His^+^–Phe. Whereas His^0^–Phe-stacked conformations are π–π in nature, His^+^–Phe can engage in very attractive cation–π interactions in which the positively charged His is attracted to the negatively charged π-system of Phe. Interactions of the cation–π type are discussed in greater detail later. Although the stacked interactions are ∼1 kcal/mol more favorable energetically for His^+^–Phe compared with His^0^–Phe, it cannot be concluded that His is exclusively positively charged in these geometries.

Interestingly, we observed that, for both His^0^–Phe and His^+^–Phe, the most favorable stacked interactions are consistently confined within geometric definitions of 60° < Tθ_2_ < 75° and 0° < *P* < 20°. This region of the map is indeed populated in the PDB data set, as shown by the darker background coloring of the His–Phe density contour gradients (Figure 2). This finding is surprising when we compare π–π interaction of commonly discussed aromatic pairs, as for the latter, these conformations are rarely observed (refer to discussion in Section S5 in the Supporting Information). Our observations of His preference to interact within stacked conformations (over other neutral aromatic pairs) are in line with an earlier, geometrical analysis of His–X pairs^28^ (where X is aromatic); however, those observations did not differentiate between possible His charge states or provide energetic insights. To determine whether this His preference is a result of its unique protonated state, we considered the average binding energies for stacked orientations in both neutral and charged cases. We found energies of −3.1 kcal/mol for His^0^–Phe, −3.2 kcal/mol for His^0^–Tyr, and −4.0 kcal/mol for His^0^–Trp (Table 1). In comparison, the conventional stacked Phe–Phe, Phe–Tyr, and Phe–Trp pairs contribute average binding energies of −3.3, −3.5, and −4.2 kcal/mol, respectively. We conclude that π–π interactions are only slightly weaker in His^0^–X compared with their Phe–X counterparts; thus, His^0^–X-stacked pairs can form stabilizing π–π interactions.

However, a different conclusion is reached when we consider the latter His^+^–X pairs, for which we found the average binding energies of His^+^–Phe, His^+^–Tyr, and His^+^–Trp pairs to be −4.1, −4.5, and −5.6 kcal/mol. Thus, the binding energies of His^+^–X pairs are on average at least 0.8 kcal/mol more attractive than those of their Phe–X counterparts, which may explain the relatively higher population of stacked His–X conformations. Overall, we conclude that pH fluctuations that trigger the protonation of a neutral histidine within stacked His–X pairs may stabilize His interactions with the aromatic residue by ∼ 1 kcal/mol. This remarkable energy gain should also be considered when calculating the p*K*_a_ of a specific His residue interacting with an aromatic residue, as we expect it should increase the propensity of His to be in its charged state (higher p*K*_a_). This will be discussed in a future section.

**Figure 3:**
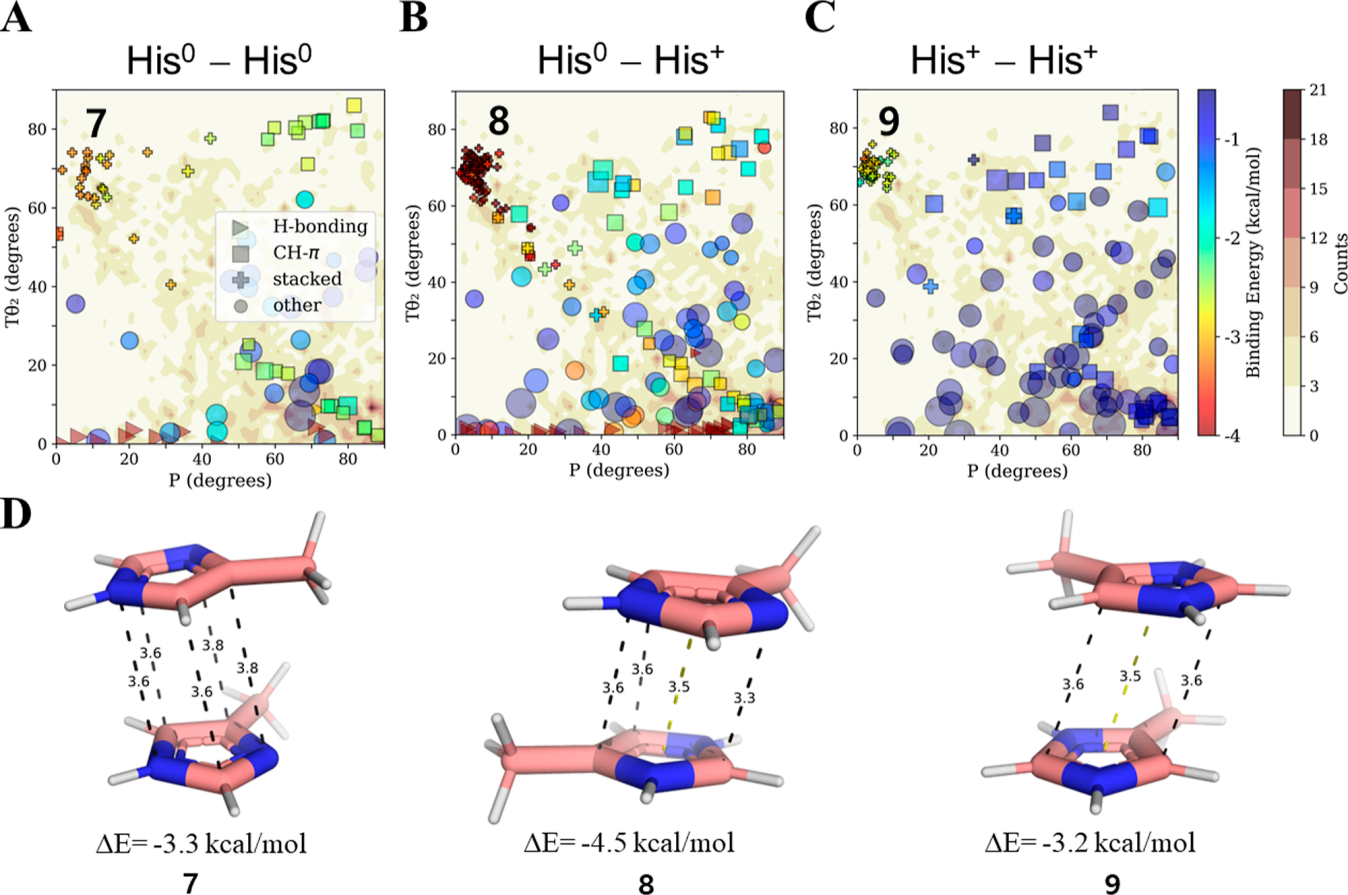
Pairwise interactions between His at different pH. The binding energies (rainbow color bar) of pairwise His–His interactions in an aqueous solvent were calculated for pairs selected from geometries obtained from high-resolution protein structures and projected onto density contour maps (white–brown color bar) calculated as described in Figure 2. Since His protonation states are unknown for most protein structures, we tested three scenarios for each of the selected pairs: (A) His^0^–His^0^; (B) His^0^–His^+^; and (C) His^+^–His^+^. Interactions between two His^0^ or two His^+^ represent high and low pH environments, whereas His^0^–His^+^ interactions may represent pH ∼ 7. The pairwise His–His interactions are categorized geometrically as H-bonding, CH–π, stacked, and other. (D). Three pairs of stacked His–His geometries (geometries **7**–**9**) possessing similar properties but different binding energies due to the His protonation state. For the symmetric case of His–His, the density values for Tθ_2_ also include Tθ_1_ in order to account for having randomly chosen one of the two His residues for the Tθ_2_ calculation.

Finally, we were interested in evaluating the effect of pH on the pairwise interaction energies in His–His pairs. For this purpose, three possible scenarios need to be considered: His^0^–His^0^, His^0^–His^+^, and His^+^–His^+^. In the sampled protein structures, we find an increased population of the stacked conformation (60° < Tθ_2_ < 75° and 0° < *P* < 20°) relative to other orientations that are expected to be populated even if only by chance (Figure 3A–C), with average binding energies of −3.0 and −3.9 kcal/mol for His^0^–His^0^ and His^0^–His^+^, respectively. This difference between the interactions of neutral and charged His is in line with our estimate of ∼ 1 kcal/mol difference for His–Phe pairs as a function of pH (Table 1). Although lowering the pH is expected to destabilize His–His conformations by favoring His^+^–His^+^, we observed that stacked His^+^–His^+^ may be favorable by up to −3.2 kcal/mol in solvated regions (Figure 3C,D). Such an attractive interaction between positively charged His pairs was previously suggested by molecular dynamics simulations in which water was the solvent,^63^ which found the more electron-deficient region around the nitrogen’s protons on one ring to be located above the complementary electron-rich region of the other ring (as per structure **9** of Figure 3D). Nevertheless, due to the similarities of the stacked geometries for His^0^–His^0^, His^0^–His^+^, and His^+^–His^+^ pairs, we cannot differentiate between the charged states of stacked His–His pairs obtained from the PDB via purely geometric analysis. This observation stresses the need to assess the effect of pH on each given pair’s energetics.

### Histidine Participates in Cation–π Interactions as either the Cation or π System, with the Former Interaction Being More Stable

**Figure 4:**
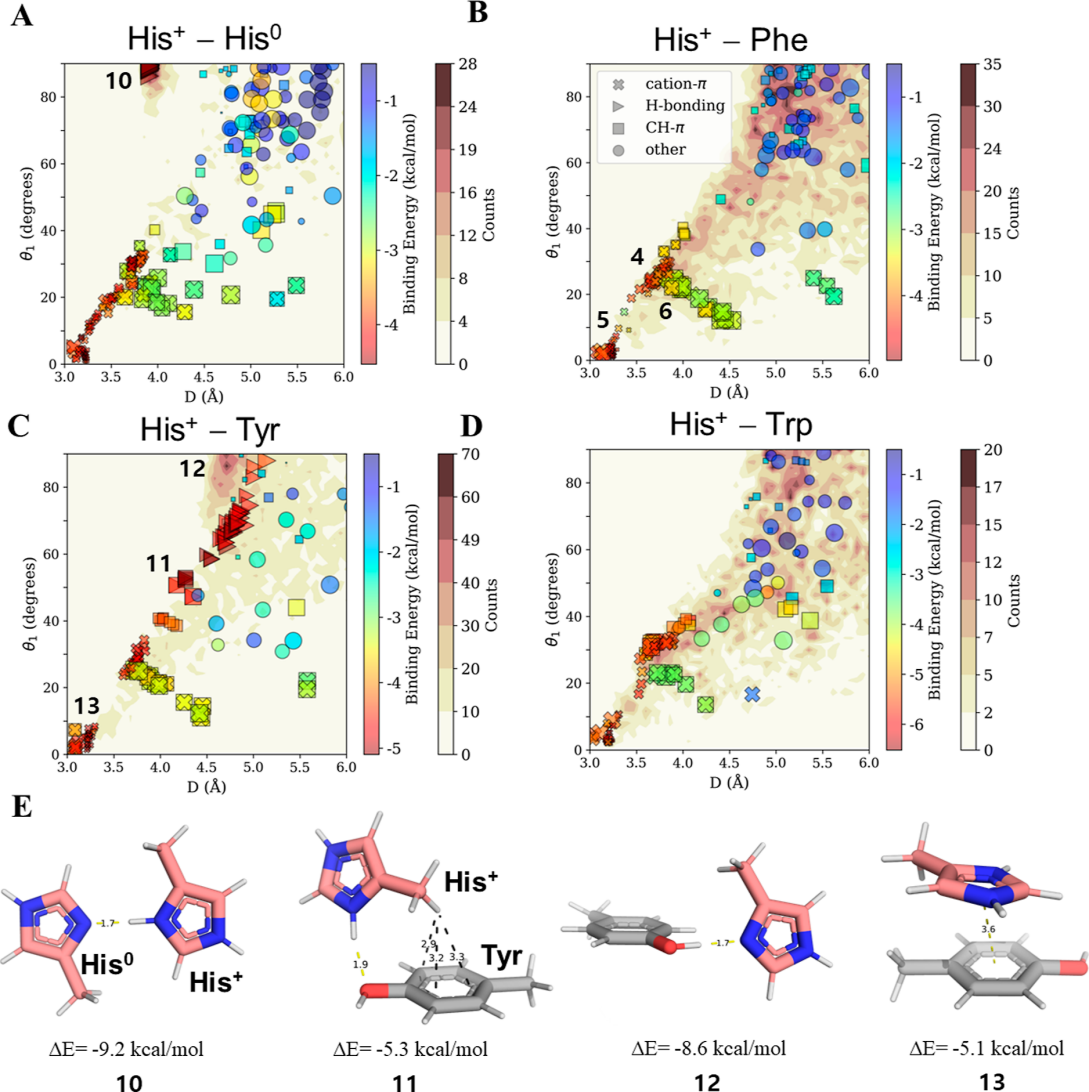
Cation–π interactions with His serving as the cation. The binding energies and geometries of pairwise interactions between His^+^ and an aromatic residue (A) His^0^; (B) Phe; (C) Tyr; and (D) Trp. The pairwise geometries sampled from the high-resolution protein structures (whose counts are indicated by the white–brown color bar) are projected along two parameters: the angle θ_1_ and the distance *D* between the center of the π system and the closest His nitrogen atom. The size of the symbol represnts the θ_2_ angle parameter. These geometric parameters were selected because they distinguish between the pairwise interactions and enable comparisons between various types of cation–π interactions involving His (see Figure 5). The interactions between His^+^ and each of the aromatic residues were studied by selected pairs that cover the projected space. All pairwise interactions were categorized geometrically as cation–π, H-bonding, CH–π, or other, and their binding energy is shown by the rainbow color scale. The numbers overlaid onto the plot refer to the His^+^–Phe geometries, which are as per Figure 2B (for geometries 4–6) and as depicted in panel E, and thus provide additional structural information in terms of parameters Tθ_2_ and *P*. (E) Four geometries (10–13) for pairwise interactions between the His cation and an aromatic residue. Geometry 10 depicts His^0^–His^+^ interactions in their lowest binding energy state, which is stabilized through H-bonds. Geometries 11 and 12 depict His^+^–Tyr H-bonds where His is positively charged or neutral correspondingly. Geometry 14 shows the most favorable cation–π configuration stabilized by CH–π interactions.

Histidine, due to its distinct p*K*_a_ value, can engage in cation–π interactions either as the aromatic π-system (paired with a cationic amino acid, such as Lys, Arg, or His^+^) or as the cation (paired with a π system, such as Phe, Try, Trp, or His^0^). It is worth noting that whereas π–π stacking interactions are fundamentally dispersive,^64^ cation–π interactions involve a positive charge interacting with the delocalized electronegative π-electron cloud of an aromatic ring.^65^ Cation–π interactions were examined using a projected coordinate system that differs from that used for π–π interactions involving His. For cation–π interactions, the pairwise geometries were projected on the distance, *D*, between the ring centroid and the proximal His nitrogen atom and the angle θ_1_ between a line normal to the aromatic ring plane and the nitrogen atom (see Figure 1F). This set of coordinates allows a comparison of all types of cation–π interactions, where the cation is either part of a ring (e.g., His^+^) or a linear side chain (e.g., Lys or Arg) (Figure 1D,E). We note that our previous discussion of His^+^–Phe using angles P and Tθ_2_ as coordinates focused on stacked orientations (Figure 2B), which are also mapped onto the new coordination scheme (Figure 4B) at *D* < 3.5 Å and θ_1_ < 10°.

Comparing all His^+^–X cation–π interactions (Table 2; without restricting the comparison to stacked geometries, refer to Section S6 in the Supporting Information) with His^0^–X π–π interactions (Table 1), where X is an aromatic residue, we observe an ∼1 kcal/mol difference favoring the protonation of His. At higher pH values, the stacked His^+^–Phe pair could lose its charge, which would weaken the binding energy of the interaction by more than 1 kcal/mol, on average. This could potentially trigger conformational adaptations in the protein to counteract this destabilization. This scenario can be further supported by a study that found that protonated His is stabilized by 1 kcal/mol relative to the neutral state in a His–Trp pair in the Barnase protein.^66^ His–Trp pairs involving protonated His were also found to contribute repeatedly to protein stability in solvent-exposed interactions within α-helices.^67^

To characterize the nature of cation–π interactions involving His^+^, we compared the interaction map of His^+^–Phe (Figure 4B), His^+^–Tyr (Figure 4C), and His^+^–Trp (Figure 4D). We observed that these interactions occur within the same conformational 2D region [distances of *D* = 3–4 Å and when the His nitrogen is directly over the center of the π-acceptor ring (i.e., θ_1_ ∼ 0–30°)]. The average binding energy of these interacting His^+^–π pair increases with increasing aromatic ring size, varying from −3.7 kcal/mol for solvent-exposed His^+^–His^0^ pairs to −5.4 kcal/mol for His^+^–Trp, with the binding energy of His^+^–Phe falling in between at −3.9 kcal/mol (Table 2). We note that, despite calculations indicating the energetic desirability of cation–π interactions in His^+^–His^0^, they are not significantly populated in the database of high-resolution PDB structures (Figure 4A), although they may be involved in stabilizing intrinsically disordered proteins that were not included in our analysis.

To verify whether the rest His’ unique His^+^–π (non His^+^–His^0^) interactions are common in PDB, we considered the type of His’ nitrogen atoms that could potentially interact with the aromatic acceptor ring (Phe, Tyr, and Trp), as shown in Figure S7. We discovered that, in these cation–π interactions, the abundance of the His ND1 atom is greater than that of the NE2 atom in the vicinity of the centroids of the Phe, Tyr, and Trp rings (Figure S7A–F). Moreover, these interactions also present more attractive binding energies when ND1 is involved. This observation is intriguing since ND1 is required to be protonated in the positively charged state of His but is usually deprotonated in the neutral state (see the Methods section), hinting at a preference for the doubly protonated His state. This implies that the charge state of His may exert a greater influence on the interaction than would be anticipated solely on the basis of electrostatic Coulombic expectations (as Phe, Tyr, and Trp are neutral), thus further suggesting that His^+^ may play an important stabilizing role in cation–π interactions at lower pH values.

To further support the preference of His to serve as a cation in cation–π interactions, we compared interactions between His^+^ and Phe with the interactions between Arg and Phe, as it is well documented that Arg participates in such interactions.^11,13,23,68^ A comparison between the strengths of the cation–π interactions formed by His^+^–Phe pairs and Arg–Phe showed that the interactions of His^+^ with Phe are even stronger than those of Arg with Phe, while the energy of the His^+^–Phe cation–π interaction is −3.9 kcal/mol, that of Arg–Phe is −3.4 kcal/mol on average (Table 2), indicating that His^+^ is an attractive cation.

**Figure 5:**
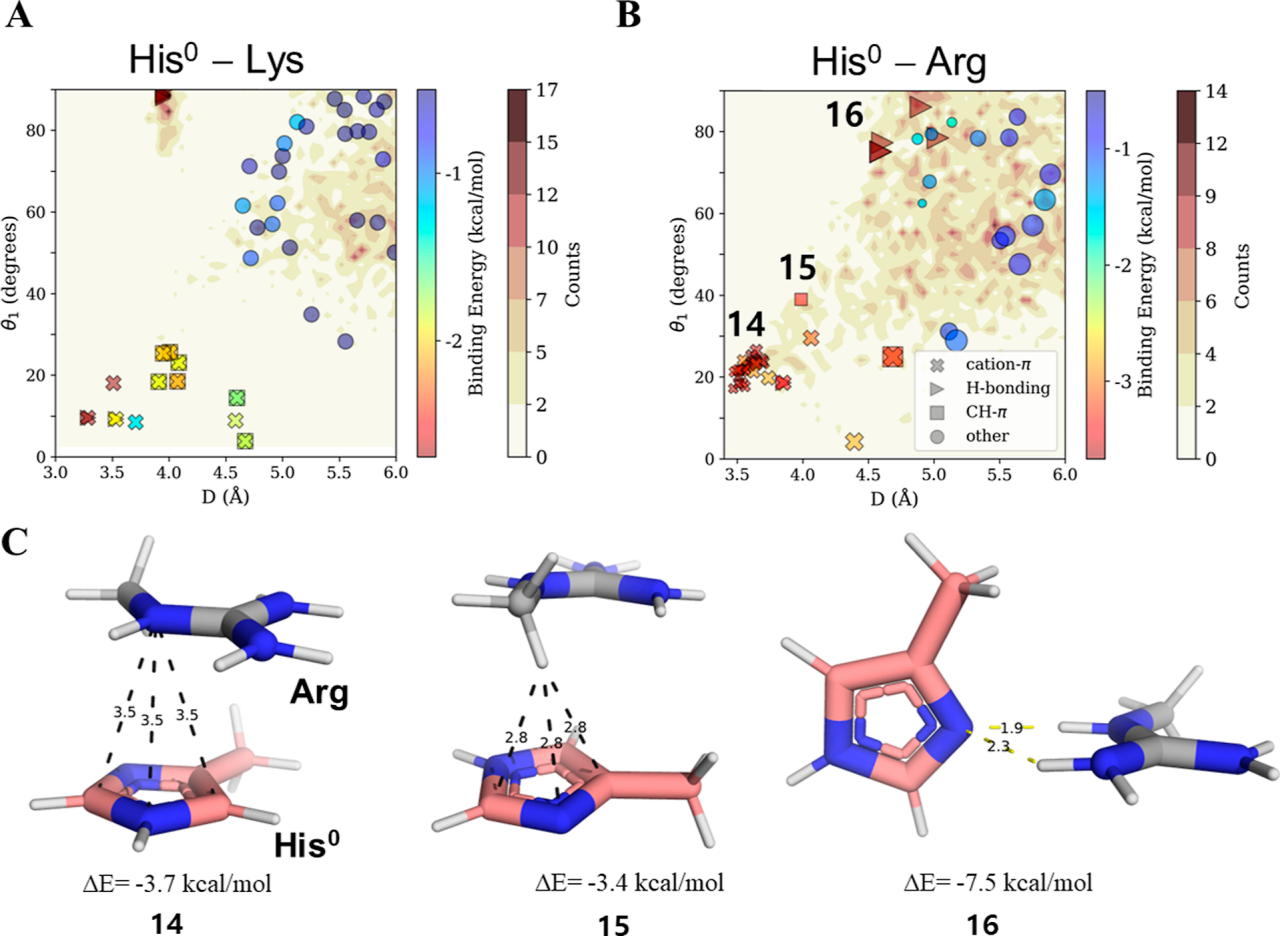
Cation–π interactions in which His serves as the π system. Binding energies and geometries of pairwise interactions between His^0^ and positively charged residues: (A) Lys and (B) Arg. The energetic and geometric analysis of the interactions between His^0^ and positively charged residues is identical to that between His^+^ and aromatic residues (see Figure 4). (C) Three geometries (14–16) of low binding energy interactions between His^0^ and Arg are depicted: geometry 14 is a cation–π interaction, geometry 15 is CH–π interaction, and geometry 16 is an H-bonding interaction.

Cation–π interactions in which His serves as the electronegative π system (His^0^) are favorable, particularly when His interacts with Arg rather than with Lys. The strength of His^0^–Lys is only ∼2.0 kcal/mol (Table 2), and such pairwise interactions are not abundant in our sampled PDB structures (Figure 5A). On the other hand, the cation–π interaction His^0^–Arg is more attractive, with an energy of ∼3.4 kcal/mol, and occurs in several protein structures (Figure 5B). The strength of the His^0^–Arg cation–π interaction is comparable with that of Arg interacting with other π systems, such as Phe or Tyr, for which the binding energies are −3.4 and −3.7 kcal/mol, respectively (Table 2). Overall, we conclude that not only are cation–π interactions involving His^+^ stronger than commonly discussed interactions in which Arg serves as the cation but also cation–π interactions in which His^0^ serves as the π system are comparable in strength with those between common π system and Arg.

### Hydrogen Bonds Involving His Are the Strongest among Aromatic Residues, with Strengths Being pH Dependent

**Figure 6:**
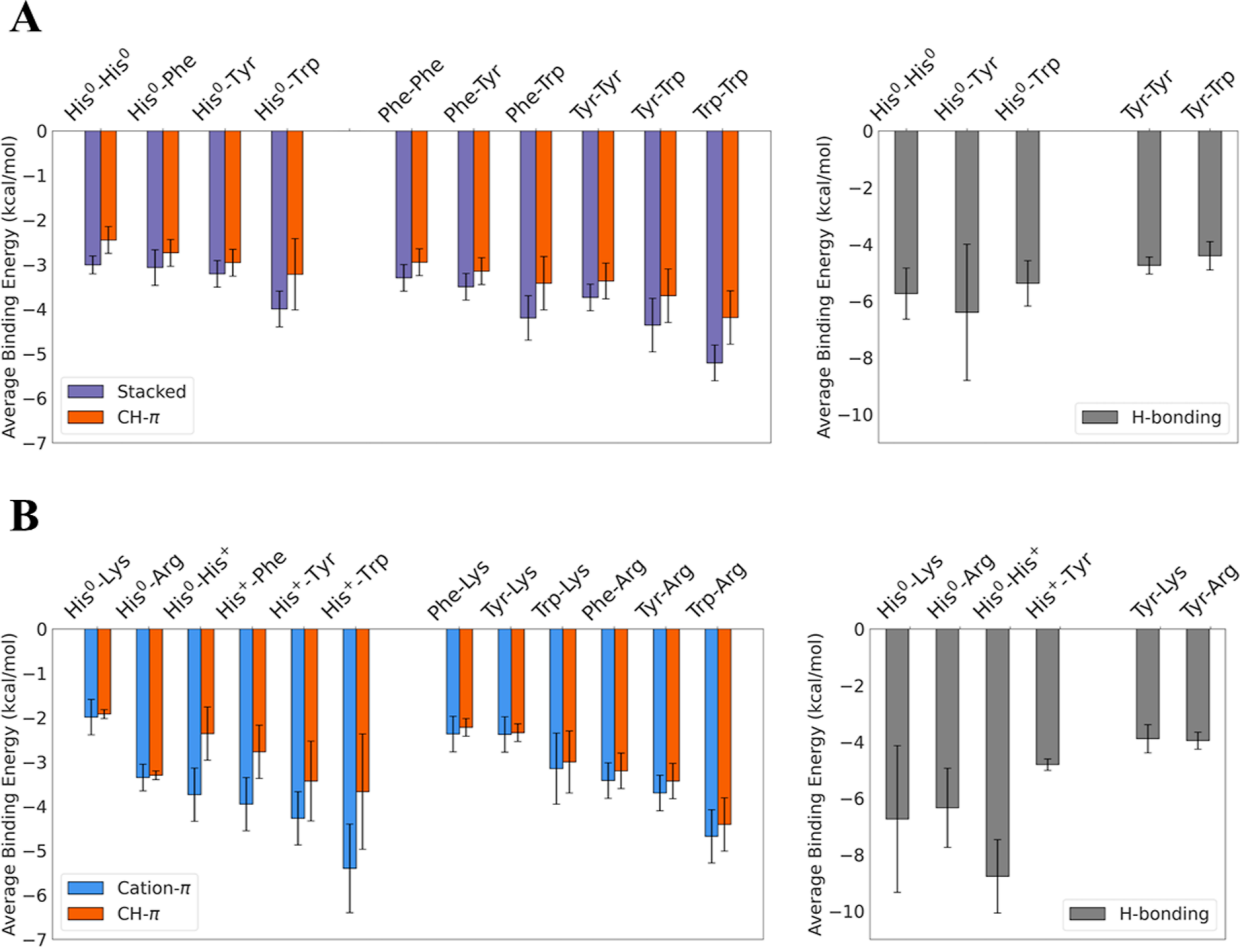
Summary of binding energies of pH-dependent pairwise interactions of His with aromatic and basic residues in solvent. (A) Interactions between His^0^ and aromatic residues (His^0^, Phe, Tyr, and Trp). The energetics of interactions between His^0^ and aromatic residues are compared with interactions between non-His aromatic residues. The interactions are categorized into three groups (based on geometric parameters): stacked (purple), CH–π (red), and H-bonding (gray). Since H-bonding interactions are possible only for some of the pairs and because of their different energetic contribution, they are shown separately. (B) Interactions between His^0^ and each of the basic residues Lys and Arg as well as between His^+^ and each of the aromatic residues His^0^, Phe, Tyr, and Trp. The energetics of cation–π interactions in which His serves as either the π-system (in cation–His^0^ interactions) or the cation in (His^+^–π interactions) is compared with the interactions of Lys and Arg with the aromatic residues Phe, Tyr, and Trp. The interactions are categorized into three groups (based on their geometric parameters): cation–π (blue), CH–π (red), and H-bonding (gray).

In addition to stabilization via π–π and cation–π interactions, His pairwise interactions can be stabilized by hydrogen bonding in the case of both protonated His^+^ and deprotonated His^0^, which are the dominant forms under acidic and basic conditions, respectively. Consequently, we sought to understand how the charge on His affects the energetic strength of hydrogen bonding as affected by pH. As both His^0^–X (X=Tyr, Trp, His^0^, or His^+^) and His^0^–Arg/Lys systems can participate in H-bonds, we compared them separately (Figure 6) with respect to the corresponding reference aromatic–aromatic pairs (i.e., Tyr–Tyr and Tyr–Trp in Figure 6A) or cationic–aromatic pairs (i.e., Tyr–Lys and Tyr–Arg in Figure 6B). In both aromatic–aromatic and cationic–aromatic systems, we observed that H-bonds that include His exceed the strength of those found in the reference pairs, suggesting that His is a strong H-bond donor and acceptor.

Assessing the effect of pH, we found that H-bonds in His^0^–His^0^ pairs (which dominate at pH >pK_a_) contribute an average binding energy of −5.7 kcal/mol (Table 1), which is an approximately 3 kcal/mol smaller contribution to binding strength than is obtained from H-bonds in His^+^–His^0^ pairs (which dominate at pH ∼pK_a_). However, an opposite trend is observed for the hydrogen bonding of His–Tyr pairs, where stronger H-bonds are formed at higher pH. The mean binding energy from H-bonds in His^0^–Tyr is −6.4 kcal/mol (Figure 6A and Table 1), whereas that between His^+^ and Tyr is −4.8 kcal/mol (Figure 6B and Table 2). The strength of H-bonds in His^+^–Tyr is comparable to those in Tyr–Tyr and Tyr–Trp pairs, which contribute −4.8 and −4.4 kcal/mol, respectively. Although the H-bonds of charged molecules are typically regarded as stronger than those formed by neutral molecules, we show a remarkable energetic advantage arising from hydrogen bonding in His^0^–Tyr interactions over that in His^+^–Tyr interactions. Our observation provides a molecular explanation for the effect of pH on the stability of hydrogen bonding between His and Tyr in the Apoflavodoxin protein^69^ and suggests that such interactions may exist in other proteins.

We found the opposing dependence of H-bond strength on pH for His–His (destabilized by increasing pH) and His–Tyr (stabilized by pH increase) interesting. This contrast may be resolved by the observation of these pairs associated with distinct regions of the density contour maps. Specifically, His^+^–His^0^ H-bonds (Figure 4A, triangles) are highly spatially restricted (*D* ∼ 4 Å, θ_1_ > 80°), whereas the H-bonds of His^+^–Tyr pairs (Figure 4C) exhibit variability in distance and elevation angles (4 Å < *D* < 5 Å; 45° < θ_1_ < 90°). His^0^–Tyr H-bonds resemble those formed by His^+^–His^0^ in their narrow restricted geometric space (Figure 4C,E geometry 12 and Figure S6). Whereas H-bonds involving Tyr (as the acceptor) occur via an aromatic ring substituent, interactions involving His^0^ occur through an aromatic ring atom acceptor and are thus constrained to a very specific in-plane planar geometry (Figure 4E geometry 10) in contrast to the case of His^+^–Tyr (Figure 4E geometries 12–13). We found that these very attractive His^0^–Tyr/His^+^ interactions are not only geometrically restricted but also very populated (Figure 4A,C, deep brown background) compared with the His^+^–Tyr case (paler background). The geometries that populate the dense H-bonding region for His^+^–His^0^ can contribute up to −9.2 kcal/mol to the binding energy (geometry 10 in Figure 4E), with His^0^–Tyr not far behind at −8.6 kcal/mol (Figure 4E, geometry 12). The most stable His^+^–Tyr H-bond contributes considerably less energy at −5.3 kcal/mol (Figure 4E, geometry 11), thus supporting the surprisingly higher abundance of H-bonds involving His^0^.

The remarkable binding energy found for H-bonded His^+^–His^0^ pairs compared with His^0^–His^0^ pairs, where the latter are comparable to typically reported H-bonds strengths, was previously studied under hydrophobic conditions, with which many His–His geometries in the PDB are compatible.^62^ To further study His–His H-bonds, we evaluated their strength in a hydrophobic environment (proxied by the gas-phase environment). The H-bonds under these conditions (Table 1) are in line with the values reported earlier.^62^ These energies may also explain our observation that cation–π relationships are sparsely populated, whereas H-bonding relationships are highly populated for His–His pairs (Figure 4A). The favorable energies of His–His interactions suggest that His prefers to participate in highly stabilizing H-bonds over stacking, cation–π, and CH–π interactions (Figure 6A). Similarly, our QM calculations predict stronger H-bonding compared with cation–π interactions for His^0^–Lys pairs, which is consistent with their much higher occurrence in the PDB data set (Figure 5A). H-bonds in His^0^–Lys pairs exhibit very restricted and relatively highly populated geometries (Figure 5A; *D* ∼ 4 Å, θ_1_ > 80°), which overlaps the region found for His^+^–His^0^ pairs (Figure 4A). This resemblance again suggests that a His–Lys proximate pair will preferentially participate in H-bonding, with this possibility restricted because of the role of His as an H-bond acceptor and the requirement that the Lys hydrogen atom be positioned in the plane of the His ring. For His–Lys, the preference for H-bonding is further supported by it contributing up to 3-fold stabilization compared with other possible interaction types (Figure 6B), where the average contribution from H-bonds is −6.7 kcal/mol, compared with ∼−2 kcal/mol for His–Lys cation–π or CH–π interactions (Table 2). His–Lys H-bonds are stronger than those formed by His–Arg pairs (Figure 6B) but also by those involving Tyr–Lys (−3.9 kcal/mol). Considering the plausible limitations of implicit solvent models, the energetic stability of H-bonds might be lower when calculated with a more accurate solvation model. Explicit consideration of discrete water molecule was previously performed for Arg side chain, suggesting a slight decrease in the binding energies.^70^ The influence of water H-bonding on imidazole (particularly for His–Lys case where His is neutral, and therefore H-bonds occur directly and are restricted through the nitrogen ring atom) should be considered in future work. Nonetheless, the low abundance of His–Lys pairs in resolved protein structures in the PDB, irrespective of their conformations, may suggest that such strongly attractive H-bonds are disadvantageous in structured proteins. However, we must note that the low abundance of His–Lys instances may also reflect the possibility of the charge on His becoming positive and thus electrostatically repelling the Lys residue. Nevertheless, we found more instances of His–Arg pairs than His–Lys pairs, despite Lys being more abundant than Arg in our database.

### His Can Participate in CH–π Interactions Regardless of Its Protonation State, but They Are Weaker than π–π and Cation–π Interactions

CH–π interactions are found to contribute to the stabilization of various pairwise interactions. As a standalone interaction, they are weaker on average than other interactions (e.g., π–π strengths) for both His-inclusive and His-exclusive aromatic–aromatic pairs (Figure 6A). For His–Phe pairs, we observed in the gas phase (Figure S8A) that the most energetically stabilizing interactions are not “pure” stacked interactions but rather mixed interactions involving specific stacked conformations with additional contributions from CH–π interactions, as shown in Figure 2C, geometry 1. Even for solvated interactions, we found that the energy of the most stabilized “pure” stacked conformation of His^0^–Phe (Figure 2, geometry 2) is the same (−3.7 kcal/mol) as that of a conformation stabilized by two simultaneous CH–π contributions (Figure 2, geometry 1).

With the exception of Trp, these CH–π are typically only slightly weaker than “pure” π–π interactions, on average (when including both stacked-stabilized and pure CH–π cases). Considering the high specificity required for π–π contacts, which are very constrained geometrically (Figures 2A,B and 3A–C), CH–π interactions occur across a broad range of angles. We found these angles correspond to the most populated region of the PDB data set for His–Phe pairs, where *P* > 30° in tilted (Figure 2, geometry 4) and perpendicular T-shaped (Figure 2, geometry 6) geometries, which is a map region that is inaccessible to the stacked conformation. Thus, while CH–π interactions are weaker than others, they may be more commonly found and are less specific.

Given the observed apparent prevalence of CH–π interactions, we proceeded to study how they are affected by pH, while considering the role of His at various Tθ_2_ values. For Tθ_2_ > 45°, an aromatic His hydrogen points toward the center of the Phe ring, where His serves as a CH donor and Phe as the π-acceptor (Figure 2IV,VI). For Tθ_2_ < 45°, the roles of Phe and His are reverse (see Figure 2III), whereas at Tθ_2_ ≈ 45°, both interaction types occur, with His and Phe variously participating as both CH donors and acceptors (Figure 2I).

When His is neutral, the binding energies of CH–π interactions in His–Phe are −2.7 kcal/mol on average (Table 1), which is not much weaker than that of the reference Phe–Phe pair at −2.9 kcal/mol. Intriguingly, even when His is positively charged (Table 1), the binding energies of its CH–π interactions mirror those of the neutral pairs with a binding energy of −2.8 kcal/mol, on average. Consequently, it seems that fluctuations in the pH have a very limited impact on the stability of CH–π interactions. This similarity may serve as a simple means for reducing the pH sensitivity of His pairwise interactions in contexts where preserving the protein local structure is important.

More surprisingly, CH–π interactions in which His serves as the hydrogen acceptor (Tθ_2_ < 45°) occur with a high count density only when His is in the neutral state by the neutral His state (Figure 2A,B). While some QM calculations predict that His^+^–Phe interactions can be formed, they diverge from the populated His–Phe pairs in proteins and exhibit relatively weak binding energies of about −2 kcal/mol. This implies that CH–π interactions, in which His functions as a hydrogen acceptor, are generally exclusive to the neutral state. This can be rationalized by the positive nature of the His^+^ ring, which appears to prevent it from partially accepting a hydrogen atom. This characteristic can potentially serve as a geometric (rather than energetic) switch controlling His functionality in response to changes in pH. Consequently, we suggest that the limitation of His-involving CH–π interactions to those in which His^0^ participates as the hydrogen acceptor should be incorporated into existing p*K*_a_ predictors so that evaluation of the p*K*_a_ of histidine can take into account not only energetic criteria but also geometric restrictions when calculating the propensity to lose a proton.

Finally, we were interested in comparing the relative strengths of His^+^ involving CH–π and cation–π interactions (Figure 6B). We observed that unlike the comparable strengths (see further discussion in Supporting Information Section S9) found for the CH–π and cation–π interactions of reference pairs involving Arg or Lys cations (paired with Phe, Tyr, and Trp), cation–π interactions involving His^+^ are much stronger than the corresponding CH–π. While some overlaps are observed for His^+^–X pairs (Figure 4A–D), CH–π interactions appear to be less frequent than cation–π interactions where His residues are involved. This trend is contrary to the common aromatic–aromatic interactions that prominently feature CH–π over π-stacking geometries (Figure 2A,B), further highlighting the importance of His acting as the cation in cation–π contacts.

### Correlation between p*K*_a_ Values of Histidines and the Geometries of Their Pairwise Interactions

His charge state can be inferred in various ways, each with its limited accuracy. For example, the charge state of His can be indicated from analysis of H-bonding patterns^28^ or from p*K*_a_ calculations.^35^ The former scheme suggests that the H-bond patterns of His with other residues side chains, backbone atoms, or water molecules may reflect its protonation state.^28^ If His serves as an acceptor, then it is deprotonated. If, on the other hand, serves as a donor in its two protonation sites, then it must be protonated. We performed such analysis to the proteins in the high-resolution data set following assignment of hydrogens to create three scenarios: each His has ND1 protonated, each His has NE2 protonated, or both sites protonated. Then, H-bonds with any atom were calculated for each scenario. Only scenarios where all cases agreed on the status were considered.

**Figure 7:**
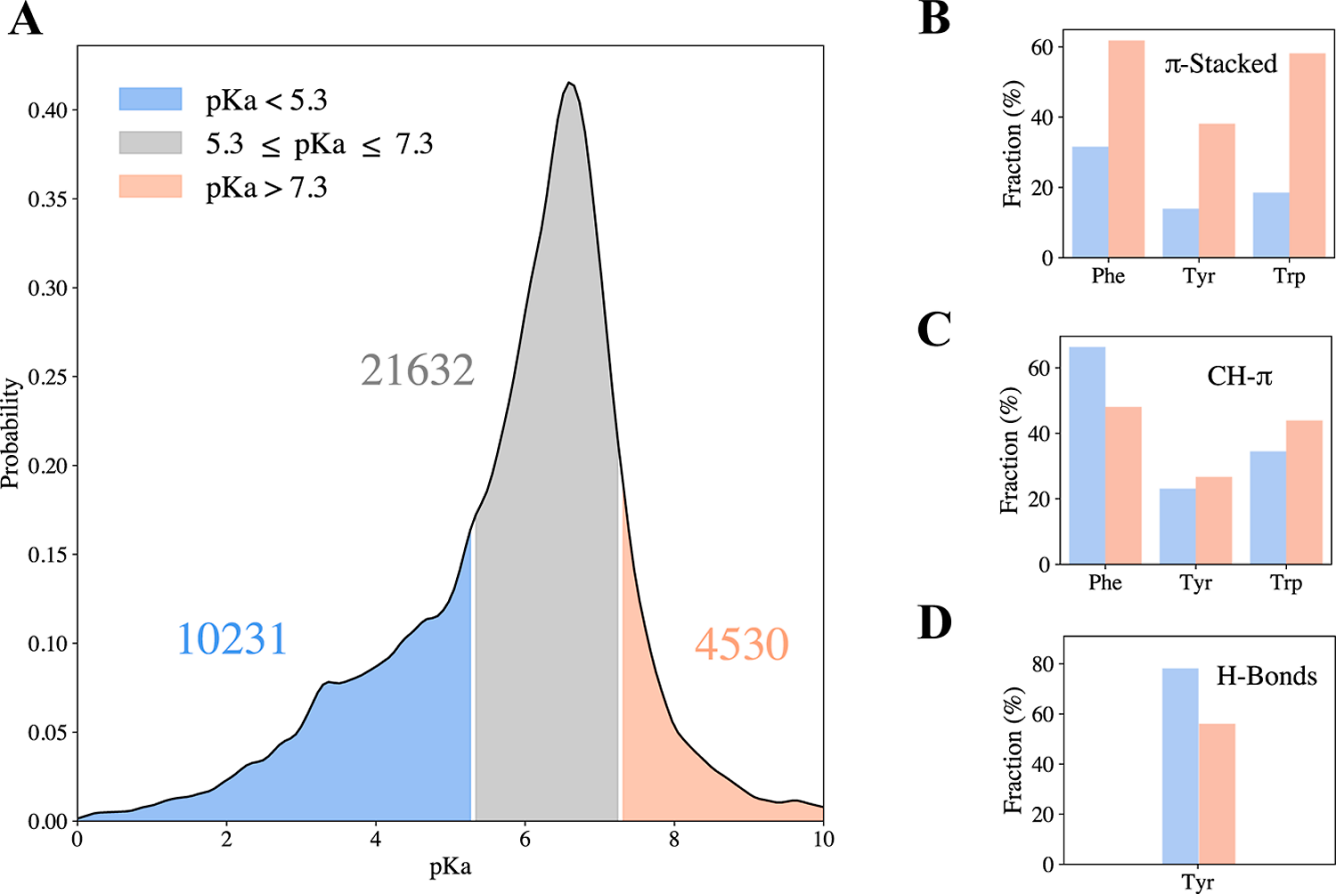
Histidine interaction type correlates with histidine-predicted p*K*_a_. (A) Distribution of p*K*_a_ values of histidine in high-resolution X-ray structures calculated using PypKA (see the Methods section). The p*K*_a_ values correspond to 36,393 His residues which are not interacting with metals. The p*K*_a_ peak value of the distribution is 6.3. The His p*K*_a_ values are categorized into three groups: low p*K*_a_ His with p*K*_a_ < 5.3 (10,231 His instances), high p*K*_a_ category with p*K*_a_ > 7.3 (4530 instances), and medium p*K*_a_ His with 5.3 ≤ p*K*_a_ ≤ 7.3 (21,632 instances). For each of the His in the three p*K*_a_ groups, all pairwise interactions between His and Phe, Tyr, or Trp were geometrically analyzed and classified as H-bonds, π-stacked, or CH–π. For each group of p*K*_a_ values of His, the occurrence of interactions is normalized independently for each pairwise interaction. (B–D) Fractions of pairwise interactions of His, depending on its predicted p*K*_a_ value, interacting with aromatic residues via (B) π-stacked, (C) CH-π geometries, or (D) H-bonds of Tyr.

Overall, the analysis of the H-bond pattern revealed 13,897 neutral His, an additional 4143 His expected to be neutral due to metal binding, and 1834 positive His. However, 52,046 His could not be determined at all based on such analysis, having only one or no H-bonds regardless of the state. An alternative way to assess the fraction of different His charge states is through p*K*_a_ calculations. To find correlations between specific His interaction types, we performed p*K*_a_ calculations to all relevant His residues in the high-resolution X-ray data set. The p*K*_a_ values of His exhibit a broad distribution with a mean of 6.3 (Figure 7A). Categorization of His p*K*_a_ into low and high p*K*_a_ groups (see the Methods section) eliminates His residues whose charge can easily fluctuate and focuses on cases where His interactions with surroundings are strong enough to affect the acidity of His. Interestingly, we found that His residues with low p*K*_a_ (i.e., likely to be deprotonated His) interact more preferably (relative to the group size) with Tyr through H-bonds compared to His residues with high p*K*_a_ (i.e., likely to be protonated His) (see Figure 7D). This observation can be supported by our current report of ∼3 kcal/mol stabilization of His^0^–Tyr compared to that of His^+^–Tyr (see Figure 6). His^+^–Tyr pairs, however, appear to participate in π-stacked interactions much more (see Figure 7B) than His^0^–Tyr pairs. Similar preference for π-stacked interactions is found for His^+^ with other aromatic residues, such as Phe and Trp. This is in line with the quantum calculations showing that in a stacked conformation, the His^+^ can be stabilized by ∼1 kcal/mol compared to the interactions with His^0^. This preference is likely to contribute to decreasing the acidity of His (i.e., characterized by a higher p*K*_a_).

Lastly, p*K*_a_ analysis can provide insights regarding the prevalence of the CH–π interaction. We found that more than 50% His–Phe interactions involved CH–π interactions (see Figure 7C), suggesting the prevalence of this often-overlooked interactions, in line with the higher density observed in the contour maps (Figure 2A,B). The higher preference of CH–π interactions for His with low p*K*_a_ than for His with high p*K*_a_ is in accordance with the quantum calculations showing restricted geometries of CH–π for His^+^ compared with His^0^ when interacting with Phe (Figure 2A,B). The relatively low preference of CH–π between Tyr and His with higher values of p*K*_a_ can be a result of the shift in the His^0^ case to prefer H-bonds over CH–π, indicating energetic driven specificity.

## Conclusions

To study the diverse interactions of histidine in proteins, we investigated the energetics and geometries of the pairwise interactions of His forms with selected amino acids that can participate in either π–π or cation–π interactions. This was achieved by exploring the interactions between His and each of Phe, Tyr, Trp, Arg, and Lys. Additionally, interactions between His pairs were investigated. To explore the dependence of these interactions on pH, some of the interactions were studied for His in both its protonation states: neutral His (His^0^) and protonated His (His^+^). Characterization of interactions between aromatic residues (Phe, Tyr, Trp, and His^0^) and His^0^ or His^+^ allows direct comparison between π–π and cation–π interactions in highly similar systems that differ only regarding whether His serves as the π system (i.e., in the form His^0^) or as a cation (i.e., as His^+^). Moreover, the choice of residues allows comparison between different cation–π interactions involving His, where His serves as either the π system (e.g., Arg–His^0^) or as a cation (e.g., His^+^–Phe). The properties of π–π interactions that His forms with other aromatic residues are compared here with other π–π pairs formed between conventional amino acid π systems.

To achieve a comprehensive analysis of π–π and cation–π interactions, each of the selected pairwise interactions was studied for all possible configurations as sampled from high-resolution crystal structures. In addition to elucidating π–π and cation–π interactions, this approach also captured other types of interactions involving His, such as CH–π interactions and hydrogen bonding, whose energetic strength is also pH dependent.

We found that His is versatile and can participate in several major types of interactions, with a strength comparable to that found for other residues that often participate in these interactions. The π–π interactions formed by His are of a similar strength to those between other aromatic side chains. Similarly, His^0^ participates in favorable cation–π interactions characterized by a similar strength to those formed between other residues. Protonated His (i.e., His^+^) serves as a better cation than Arg and Lys in terms of the strength of its cation–π interactions with the same aromatic residue. In particular, a comparison of the bonding interaction strengths of cation–π interactions in which His serves as the cation (His^+^) compared with when His serves as the π system (His^0^) revealed stronger interactions in the former case. Since cation–π interactions involving His^+^ dominate under acidic conditions, whereas those involving His^0^ dominate under basic conditions, these findings suggest that although His can participate in cation–π interactions under conditions of low and high pH, the strength of these interactions is greater under acidic conditions.

Our survey of His interactions with aromatic and basic residues shows several instances in which pH changes modulate His interactions. A clear difference is observed in the effect of pH on the strength of His stacking interactions with aromatic residues. Stacking interactions involving His may have biophysical importance, as they are more frequently found in protein structures when His is involved, whereas stacking interactions are less common when two non-His aromatic residues are involved. We observed that π–π interactions between His^0^ and an aromatic residue exhibit substantial geometric overlap with those of cation–π interactions formed between His^+^ and an aromatic residue, with the latter being more stable by about 1 kcal/mol. The greater stability of interactions between protonated His and aromatic residues (via cation–π interactions) compared with neutral His and aromatic residues (via π–π interactions) has previously been observed for His–Trp pairs.^66^ This difference in the energetic strength of His interactions with aromatic residues depending on the His protonation state implies that His supports a conformational transition upon a change in pH. The sensitivity of His interactions to pH has further facets. For example, the strength of H-bonds formed with His may depend on the pH, particularly when formed between His and Tyr, with a bias toward deprotonated His. CH–π interactions involving His also depend on pH, particularly when it acts as the hydrogen acceptor rather than the donor. When His serves as a hydrogen acceptor, CH–π interactions are accessible only to His^0^ but are obscured for His^+^, again supporting alternate His conformations upon pH change. This scenario is particularly intriguing given the observed prevalence of CH–π interactions involving His in structured proteins.

The versatility of His not only manifests upon changes in pH but also under conditions of constant pH and especially under physiological conditions where the probability of histidine being either His^0^ or His^+^ can be shifted simply by the chemical environment of the protein. Determination of the protonation state of His under such conditions is of high importance; yet, the power of the available tools is limited. The current study provides some indirect insights. For example, the stronger stacking interactions of aromatic residues with His^+^ compared with His^0^ may support higher p*K*_a_ values for such His. At pH ≈ p*K*_a_, the population of both His protonation states is more probable, increasing the likelihood of His^0^–His^+^ interactions, which are found here to form strong cation–π interactions and to engage in strong H-bonding. Pairwise His–His interactions are found to be relatively poorly populated in proteins structures. It is possible that there is a natural selection against such strong interactions between His^0^–His^+^ (which is also valid for His–Lys). Nevertheless, we note that our bioinformatic survey includes only high-resolution protein structures, so the possibility that such stable interactions with His may play a more significant role in IDPs cannot be excluded.

In summary, our study offers new insights into how the protonation state of His affects its intermolecular interactions with other amino acids, enhancing our understanding of its role as a molecular switch activated by a change in pH. The findings presented here could significantly refine the parametrization of His interactions in computational models and provide valuable data for improving current p*K*_a_ predictors, allowing for a more nuanced analysis of the propensity of His to adopt specific states depending on its environmental context. Additionally, quantifying the energetics of Histidine interactions might be useful for designing new materials and, in particular, Histidine-based drugs, whose specificity is dictated by the pH of the target cell.^71^

## Supporting Information Available

Atomistic details of the compounds calculated; higher level QM validation of the QM method employed for representative pairs of amino acids; estimation of error in determining binding energies as a function of the relative orientation of selective pairs; geometrical parameters used to categorize the interaction types π–π, CH–π, cation–π, and H-bonds; comparison of other π–π pair (exclusive of His) maps; abundance of selected atoms of His with respect to a partner aromatic ring center of Phe, Tyr, and Trp; and gas-phase interaction maps for His–Phe pairs (PDF)
