# Supplementary material for: Histidine in Proteins: pH-Dependent Interplay between π–π, Cation–π, and CH–π Interactions
Source: J Chem Theory Comput. 2024 Jul 22;20(15):6930–45. doi: 10.1021/acs.jctc.4c00606 (PMC11325542; doi:10.1021/acs.jctc.4c00606)
Supplement: Supplementary file 1 — ct4c00606_si_001.pdf [file ct4c00606_si_001.pdf]

Supplementary Information

Histidine in Proteins: pH dependent interplay between π–π, cation–π, and CH–π interactions

Rivka Calinsky and Yaakov Levy*

Department of Chemical and Structural Biology

Weizmann Institute of Science

Rehovot, 76100, Israel

*Corresponding author: Yaakov Levy, Department of Chemical and Structural Biology, Weizmann Institute of Science, Rehovot, 76100, Israel; email: Koby.Levy@weizmann.ac.il; Tel: 972-8-9344587

S1 Atomistic details of amino acids subjected to QM calculations

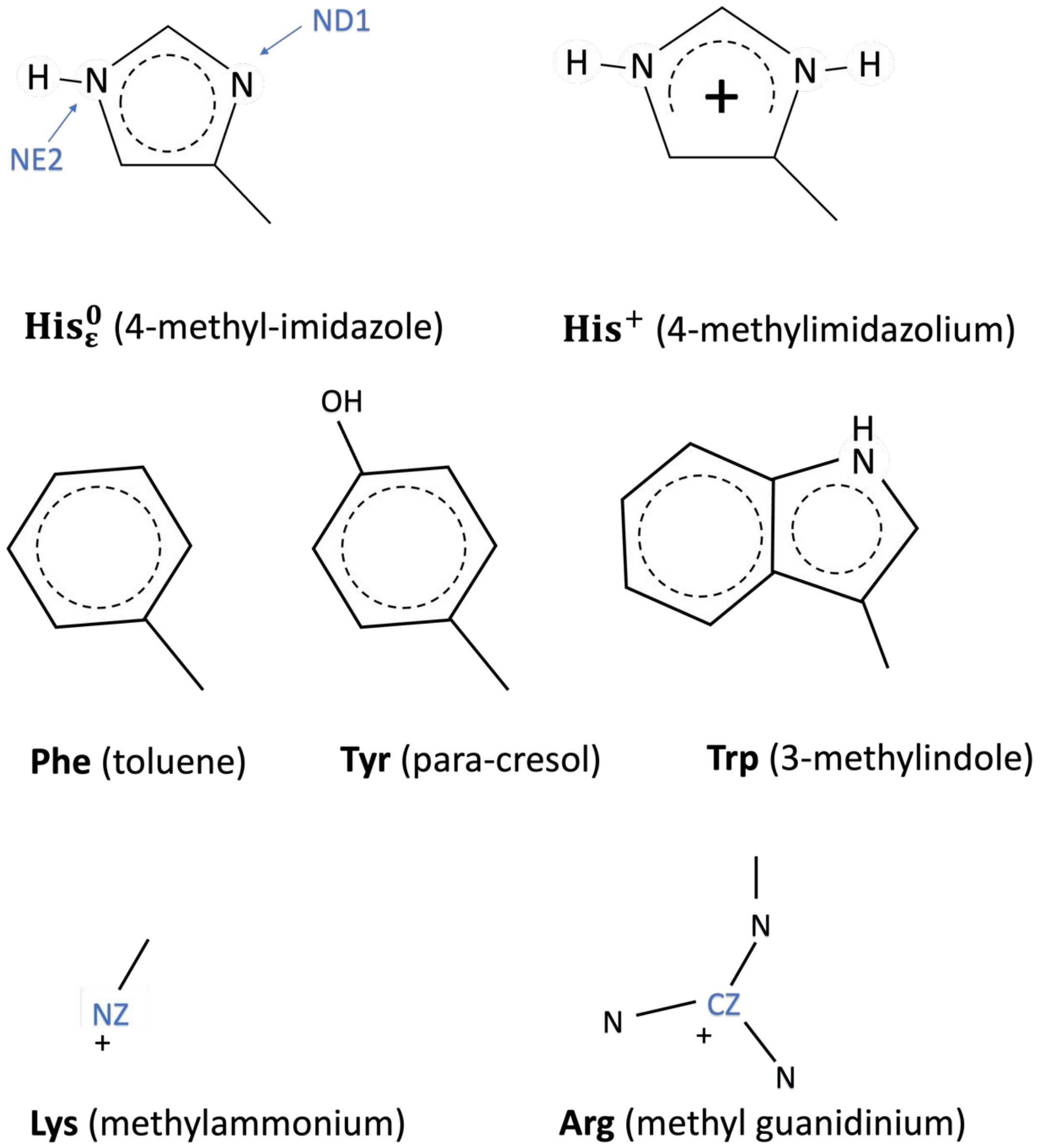

Figure S1. Schematic representations of side chains of amino acids discussed in this study. Blue atoms represent the location of the atoms mentioned in Fig. 1 (main text) as per the PDB naming convention. The corresponding chemical structures used for QM calculations are indicated and referred to as His^0, His$^+$, Phe, Tyr, Trp, Lys, and Arg.

S2 Higher level validation of the QM method employed in this work

Table S1: **Validation of the used QM method against the higher level LNO-CCSD(T).** The LNO-CCSD(T) calculated binding energies ($\Delta \boldsymbol{E}_{\boldsymbol{LNO-CCSD(T)}}^{\boldsymbol{half}}$) using a 'half-counterpoise' method for accuracy [1], derived from both 'raw' and 'Counterpoise-Corrected' (CP) methods to account for Basis Set Superposition Error (BSSE). This method was chosen due to its high performance in terms of accuracy for noncovalent interactions [2]. LNO-CCSD(T) values were calculated using vTight threshold for accuracy, satisfying $\Delta E_{LNO-CCSD(T)}^{half} = \frac{\Delta E_{CP} + \Delta E_{raw}}{2}$, where ΔE_{raw} corresponds to the energy difference of the optimized pair and the separately optimized residues. The ΔE_{CP} corresponds to the difference in energy of the optimized interacting residues and each of the residues where its partner residue is present as a 'ghost' atom (with its basis functions yet no electrons included). The $\Delta E_{revDSD-PBE86-D4}^{raw,\ def2-qzvppd'}$ error of the binding energies correspond to the error from the LNO-CCSD(T) level where the revDSD-PBE-D4 energies were calculated using 'raw' method. The last column corresponds to the fractional error from the higher-level method: $\frac{\Delta E_{revDSD-PBE86-D4}^{raw,\ def2-qzvppd'} - \Delta E_{LNO-CCSD(T)}^{half}}{\Delta E_{LNO-CCSD(T)}^{half}} \cdot 100\%$

**Index**	$\Delta \boldsymbol{E}_{\boldsymbol{LNO-CCSD(T)}}^{\boldsymbol{half}}$	$\Delta \boldsymbol{E}_{\boldsymbol{revDSD-PBE86-D4}}^{\boldsymbol{raw,\ def2-qzvppd'}}$	$\Delta \boldsymbol{E}_{\boldsymbol{revDSD-PBE86-D4}}^{\boldsymbol{raw,\ def2-qzvppd'}}$
	**Energy [kcal/mol]**	**Error**	**%Error**
1	-4.3	0.1	-3.4
2	-3.4	0.1	-3.3
3	-3.3	0.1	-2.3
4	-11.8	0.0	0.0
5	-11.3	0.2	-1.7
6	-9.6	0.1	-1.5
7	-3.8	0.1	-3.3
8	-11.7	0.2	-1.8
9	-6.1	0.1	-1.5
10	-11.9	0.1	-1.3
11	-16.8	0.3	-2.0
12	-10.8	0.2	-2.0
13	-4.2	0.1	-2.7
14	-9.7	0.0	0.1
15	-27.5	0.4	-1.6
16	-9.4	0.1	-1.4
17	61.3	0.2	0.4
18	-26.9	0.4	-1.5
19	-9.3	0.3	-3.7
	**Average**	**0.2**	**-1.8**

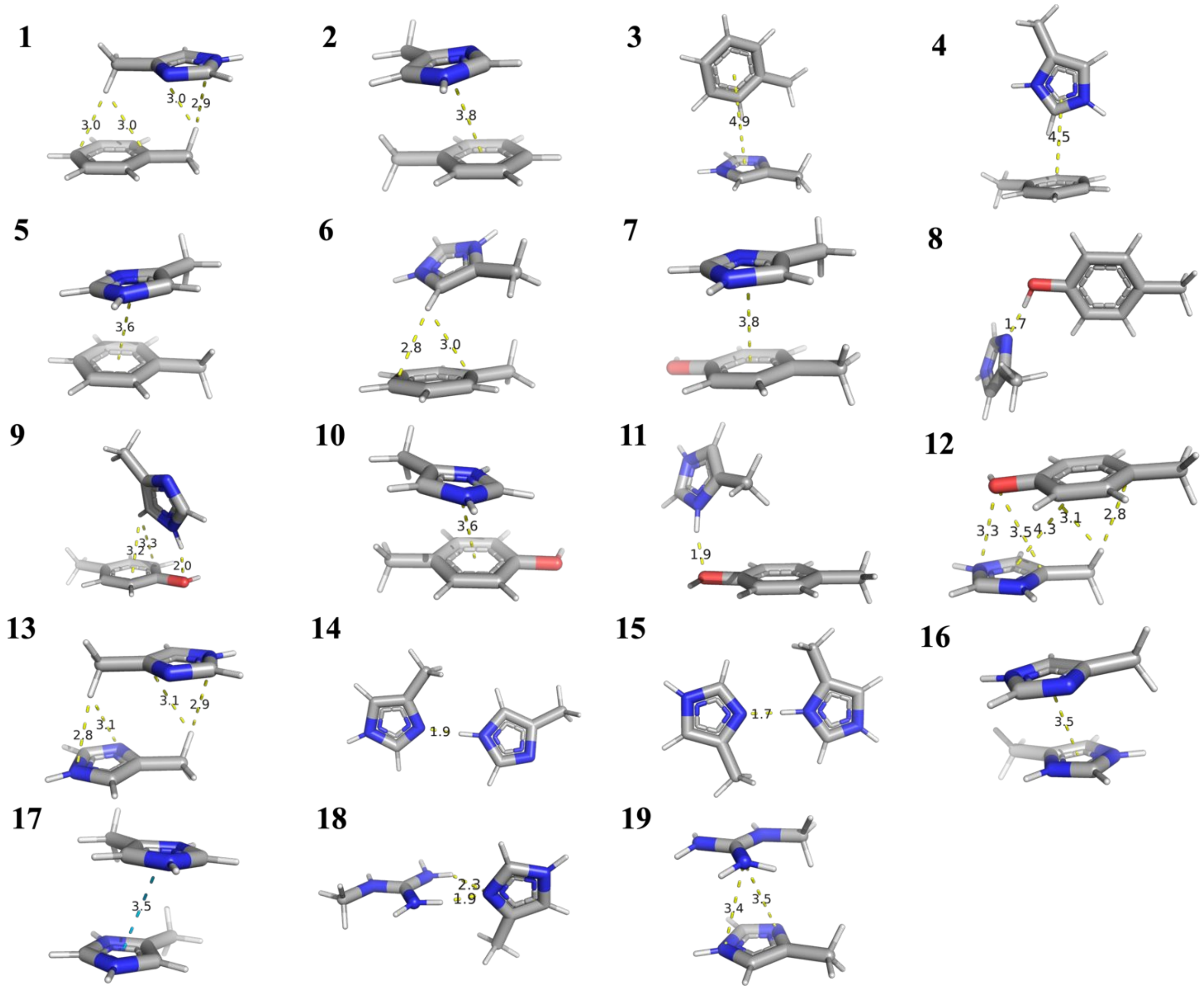

Figure S2. Representative geometries used for QM method validation. The structures are represented according to their index as shown in Table S1.

S3 Binding energy as function of relative orientations

To estimate errors that result from small changes in orientations of each pair of residues, we projected selected pairs on different coordinates employed in this study (see main text **Fig. 1**). For aromatic-aromatic pairs the relative orientation elevation angles were found to cluster our conformations into specific and meaningful interaction types (see **Fig. S3** below). We observe that as a general trend, within a selected clustered region (proximate in terms of coordinate space) the energies should not vary much relative to their mean energy. For example, His^0-Lys

(**Fig. S3A**) H-bonded group ($3.8 \leq D \leq 4.2\dot{A}$, $\theta_1 > 80°$ with an s.d <1 kcal/mol for -8.2kcal/mol (~10%). Larger deviations in one or more coordinates may yield to a larger deviation due to completely different interaction types or the presence of two or more overlapping interactions. For instance, for His^0-Phe (**Fig. S3C**) transition from $T\theta_1 \sim 50°, T\theta_2 \sim 30°$ to $T\theta_1 \sim 70°, T\theta_2 \sim 20°$ can result in a 10% lower average energy due to the transition from overlapping stacked and CH-π to pure CH-π. The only exception with higher deviations is due to very specific orientations spanned by H-bonding as can be seen for His^0-Tyr (**Fig. S3E**) and His$^+$-Tyr (**Fig. S3F**).

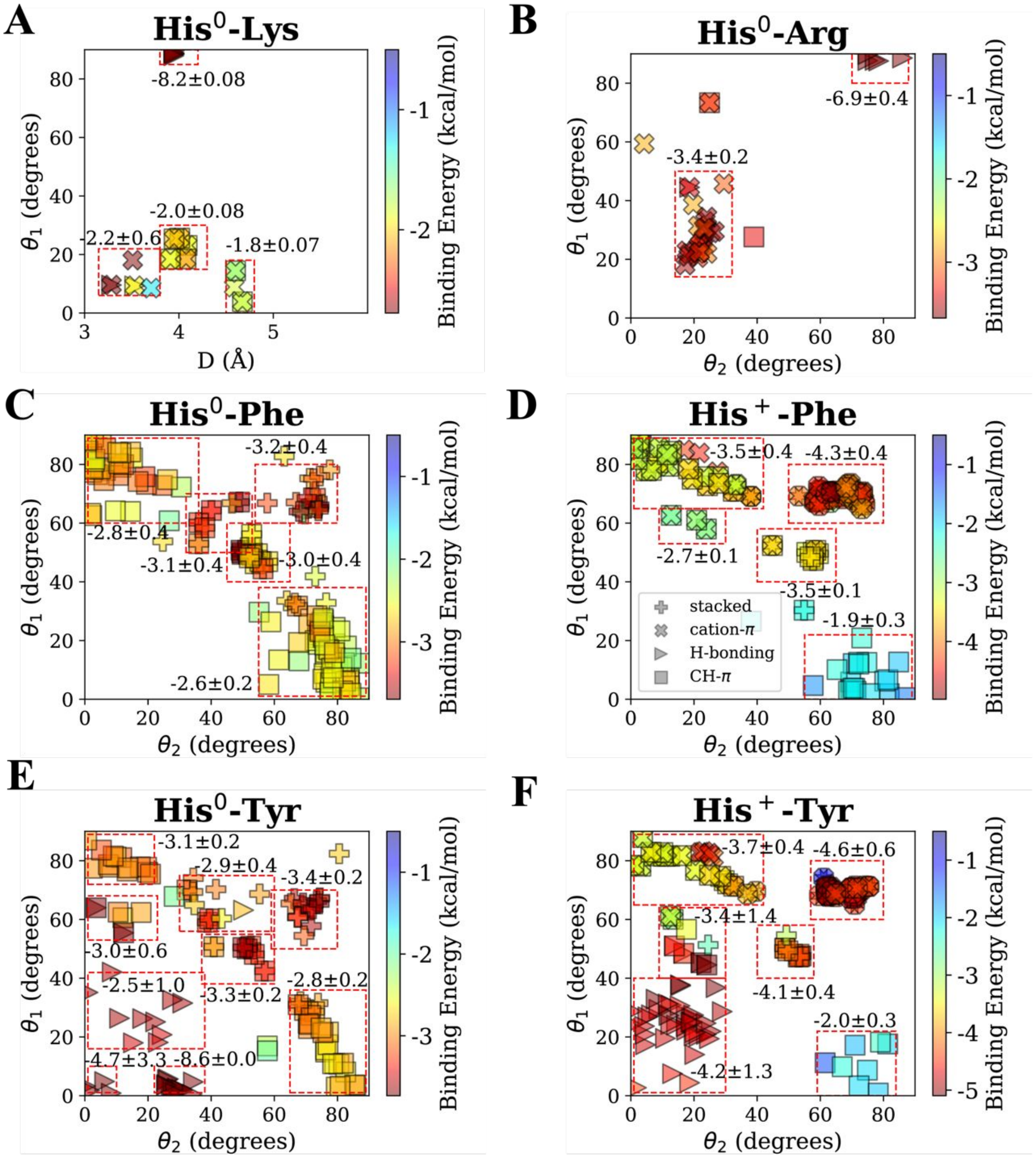

Figure S3. Estimation of the binding energy error with respect to minor changes in the fixed geometries. Mean values and the standard deviation of selected bounded regions (dashed rectangles) are noted for data points projected on two spatial coordinates. For further details refer to the description in Fig. 2 & Fig. 4 (main text). A) For His^0 -Lys pairs B) His^0 -Arg C) His^0 -Phe D) His^+ -Phe E) His^0 -Tyr F) His^+ -Tyr.

S4 Geometrical categorization of interaction types inclusive of His

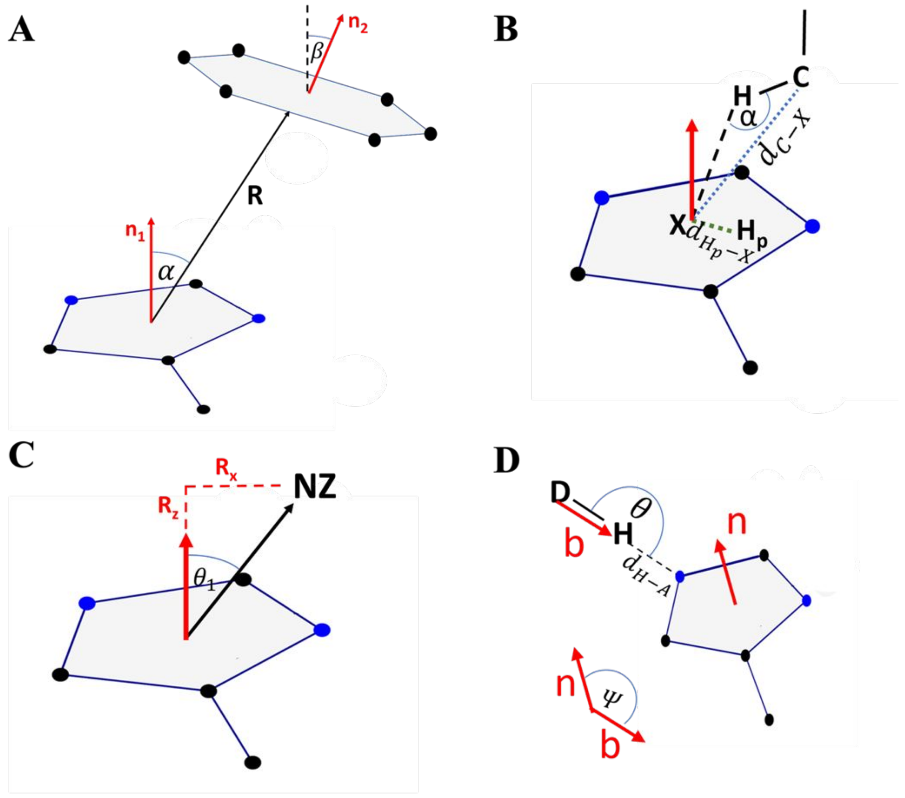

Figure S4. Selected geometrical parameters to classify the four interaction types discussed in this study: π- π, CH- π, Cation- π and H-bonds.

(A) Stacked interactions defined by $R < 5\dot{A}$, $\alpha < 45°$, $\beta < 45°$, where R is the centroids distance of two aromatic rings, α is the angle of R from the first ring's normal $\hat{n}_1$ and β is the angle of the second's ring's normal $\hat{n}_2$ from $\hat{n}_1$. (B) CH-π interaction pairs are chosen according to the Brandl-Weiss system. These are defined with $d_{C-X} < 4.5\dot{A}$, $\alpha > 120°$, $d_{Hp-X} < 1.2\dot{A}$,

where d_{C-X} the distance of the closest carbon from the centroid of the aromatic ring, X. d_{Hp-X} is the distance between the closest Hydrogen and the centroid in the ring's projection in the ring's plane (only horizontal component), and α is the angle at the closest Hydrogen atom, used to exclude overlapping π -stacking interactions. (C) Cation- π interactions. Including distance satisfying D ≤ 6Å from the center of positive charge (NZ for Lys, CZ for Arg, NE2 or ND1 for His) and the center of the aromatic ring of its pair. Additionally, the projection of this distance on the aromatic ring plane (horizontal component Rx) should satisfy $Rx \leq 2.3\text{Å}$ (D) Hydrogen-bonding. Characterized based on the definitions presented in Baker's work for sp2, for H-bonds distances of $1.4\dot{A} \leq d \leq 2.1\dot{A}$ we chose structures whose angle at the hydrogen atom (ϴ) satisfies $110° \leq \Theta \leq 180°$, and their angle at the acceptor atom Ψ satisfies $70° \leq \Psi \leq 180°$. Similarly, H-bonds for larger distances are screened within: $2.1\dot{A} < d = 2.8\dot{A}$, $80° \leq \Theta \leq 180°$, $60° \leq \Psi \leq 160°$.

S5 His' pairs prefer π-π interactions compared to other aromatic pairs

We note our observation of relatively populated stacked interactions within His^0–Phe pairs (**Fig.2** in the main text) is in contrast to observations of the reference pairs involving the commonly discussed aromatic amino acids Phe, Tyr and Trp, whose sidechains are uncharged. For these pairs, π–π conformations were infrequently observed in our PDB datasets (Fig. **S4**). As, from a statistical perspective, these geometries are expected to be rare, their overrepresentation in His–Phe (compared with Phe–Phe) may suggest that His plays an important role. We therefore also examined the interactions of His–Tyr and His–Trp (see Supporting Fig. **S5**). We found an increasing relative population in the stacked region of the density contour map for His–Tyr pairs and particularly for His–Trp, suggesting that His has a greater preference to engage in stacked orientations compared with other aromatic residues.

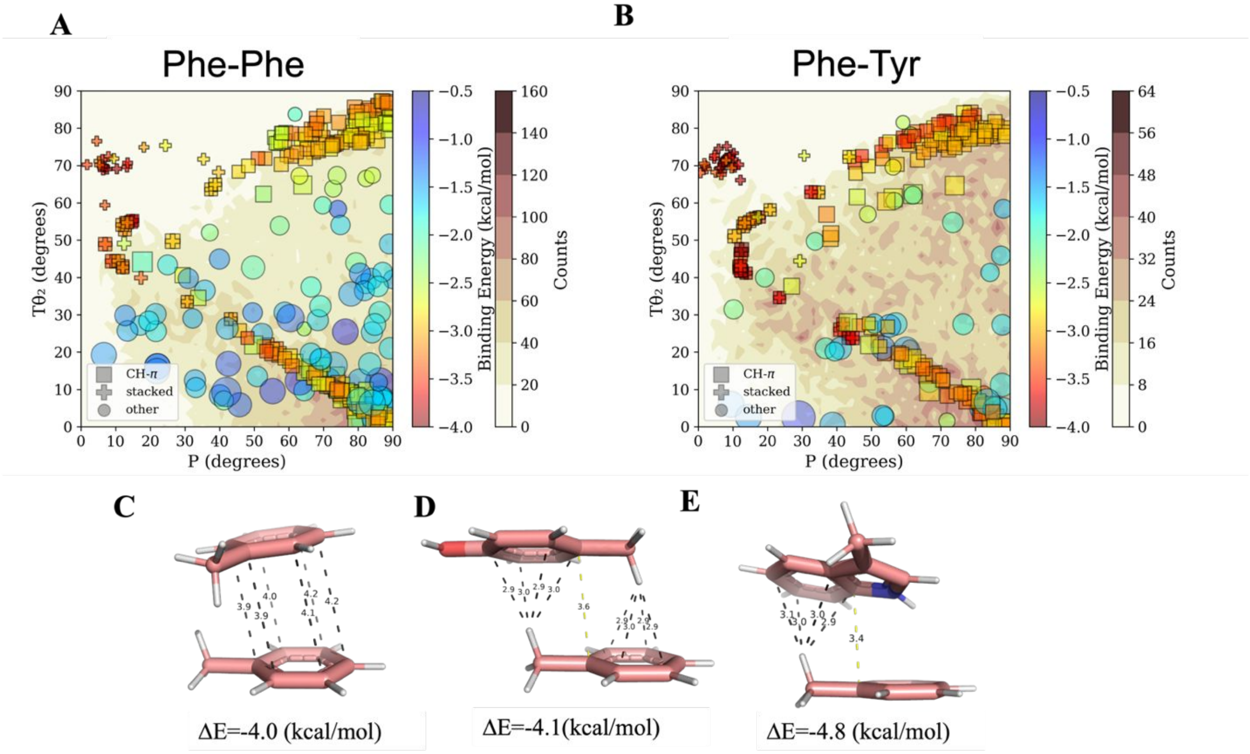

Figure S5. π- π interactions involving Phe as reference in aqueous solvent. As described in Fig. 2 (main text) the binding energies (rainbow colorbar) of pairwise Phe interactions calculated for selected geometries and mapped onto a density contour map (white-brown colorbar). (A) For Phe-Phe pairs; and (B) Phe-Tyr pairs. The π- π geometries with minimal binding energies for (C) Phe-Phe; (D) Phe-Tyr; and (E) Phe-Trp.

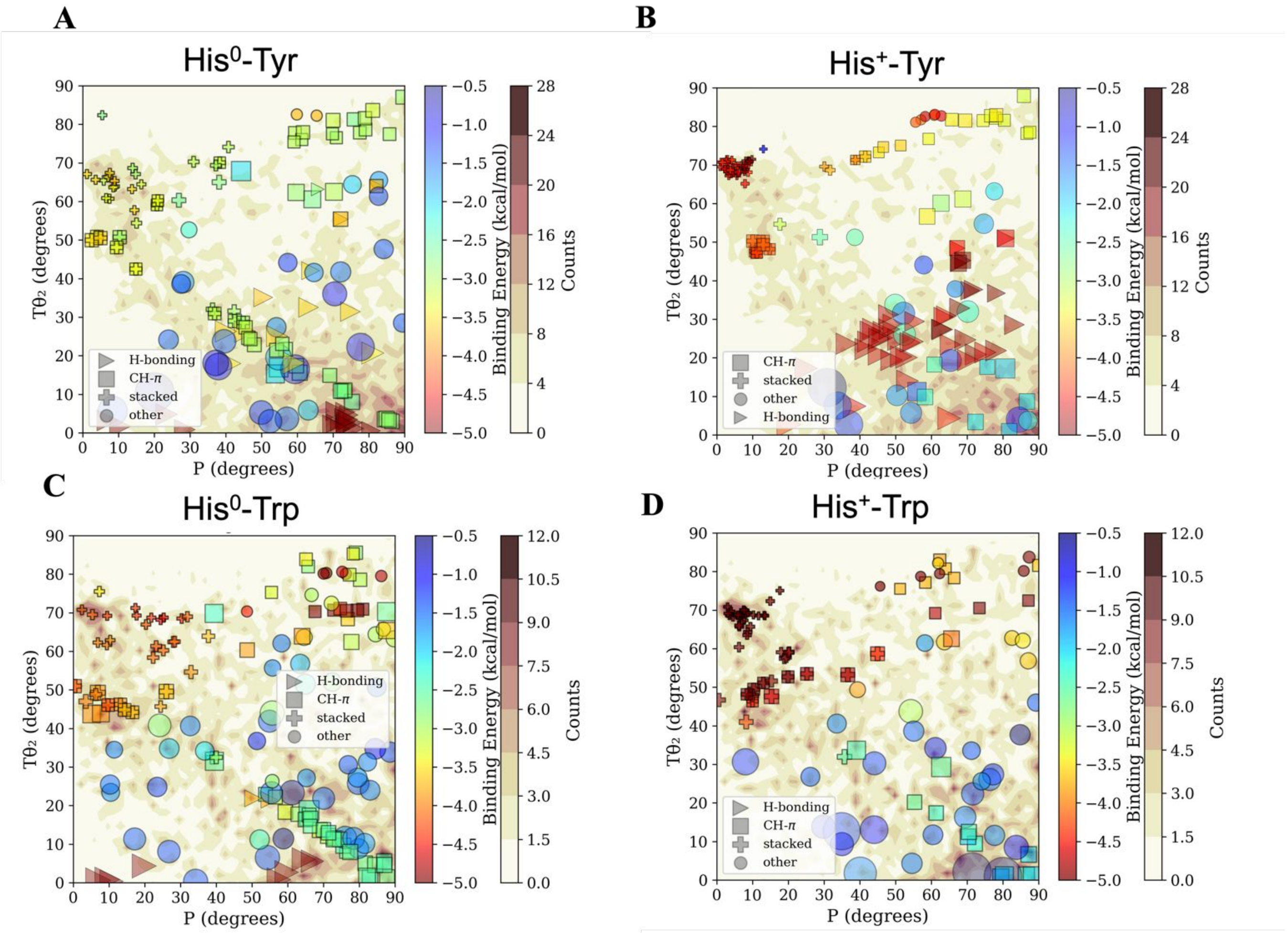

Figure S6. π- π interactions of His^0 compared to His$^+$ cation-π involving the same aromatic partners: (A) His^0-Tyr; (B) His$^+$-Tyr; (C) His^0-Trp; and (D) His$^+$-Trp. For further details refer to the description in Fig. 2 (main text).

S6 Cation-π conformations that are non-restricted to 'stacked' orientations are more common yet less stable

Our discussion in the main text Results and Discussion section was focused on 'stacked' orientation. However, **Figure 4** (main text) shows that for these stacked conformations, the optimized geometries deviate from their PDB density, especially when compared with other His–Phe conformations. Other cation–π orientations of His$^+$–Phe, namely tilted and perpendicular orientations (**Fig. 2C,** geometries 4 and 6), are confined to a similar geometric space (D = 3.5–4

Å and θ_1 ~ 20–30°). These are found in a density contour region that appears to be more frequently populated, however, it is not possible to differentiate between the contributions of similarly interacting His^0–Phe pairs within the same geometric space. Nevertheless, stacked His^+–Phe conformations are the most energetically favorable with binding energies of up to -5 kcal/mol (**Fig. 2C**, geometry 5) compared with a mere -3.7 kcal/mol for non-stacked conformations (**Fig. 2C**, geometry 6).

S7 Preference of ND1 atom to form more attractive and frequent interactions with aromatic rings

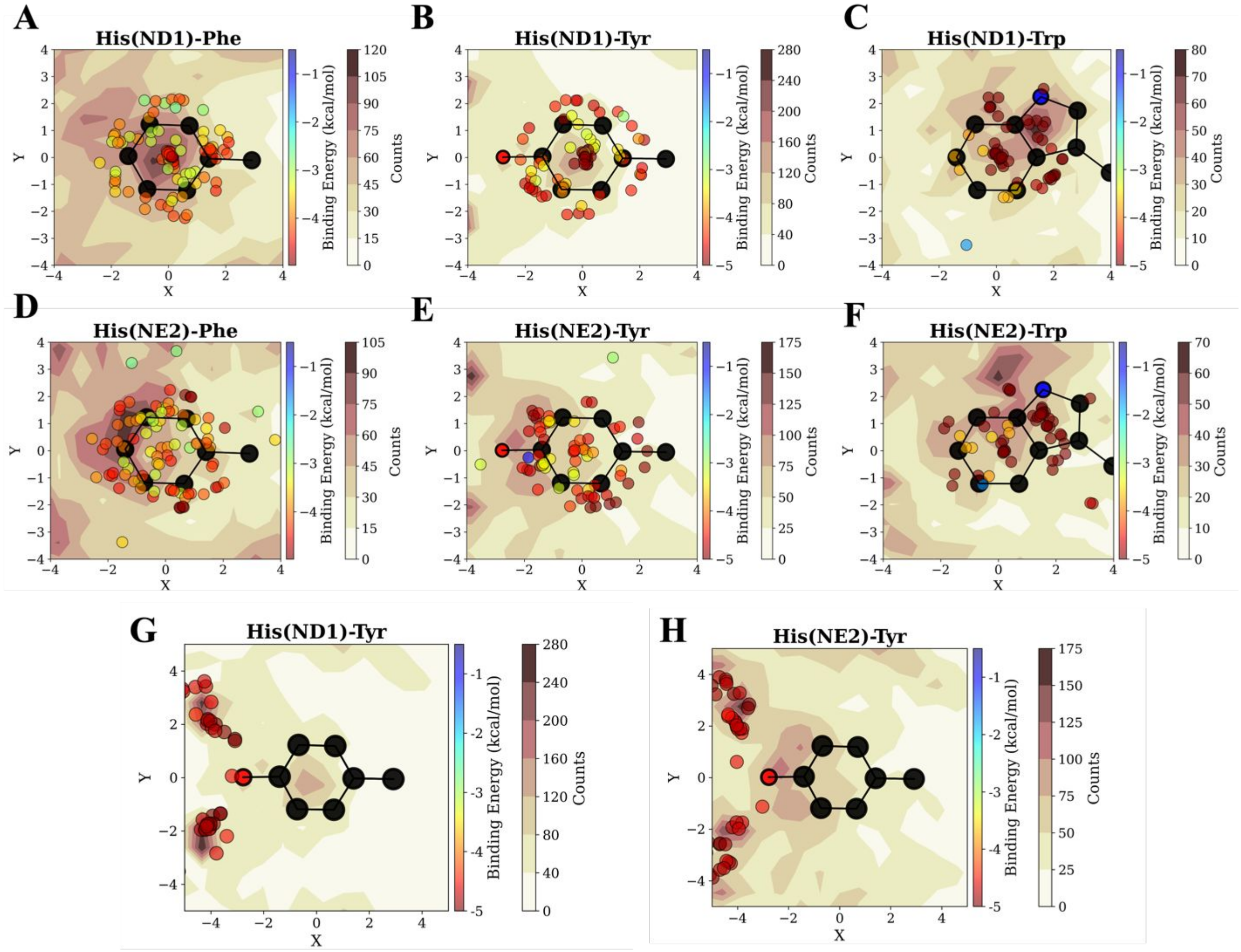

Figure S7. The nature of cation-π interactions involving His^+ in aqueous solvent.

Binding energies (rainbow colorbar) of pairwise His involving pairs mapped onto density contour gradients created by projecting the coordinates (on xy plane) of ND1 or NE2 His' atoms with respect to the center of His' aromatic partner (Phe, Tyr or Trp) as the origin, where aromatic ring plane defines the XY plane. The map shows the positions of the ND1 atom (see Fig. S1) of His relative to (A) Phe (B) Tyr (C) Trp. Positions of NE2 atom with respect to (D) Phe (E) Tyr (F) Trp. H-bonds of His^{+} in water with respect to Tyr for the His atom (G) ND1 (H) NE2.

S8 Gas-Phase interactions support CH- π interactions can be comparable or even exceed stacked conformations binding energies

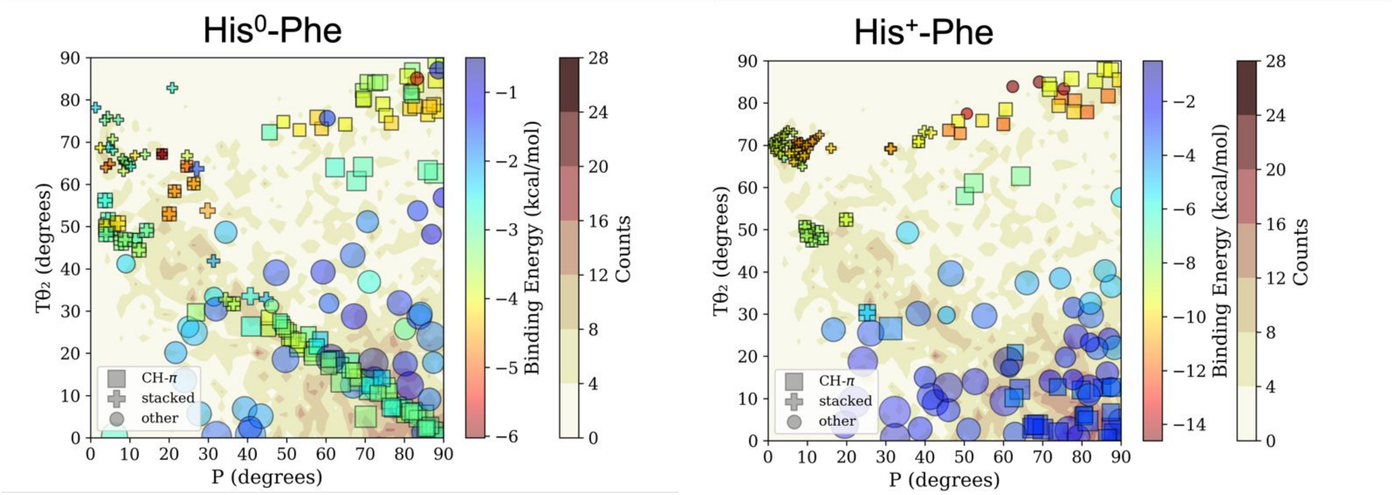

Figure S8. Gas-phase π- π interactions of His^0 compared to His^+ (A) For His^0 -Phe pairs; (B) His^+ -Phe pairs.

S9 CH- π interactions can be comparable to cation- π

The observation that stabilization is achieved via co-occurring CH–π and π–π interactions is not limited to the aromatic–aromatic case but is also found in the His–Arg case (**Fig. 5B**). We found that for cation–π pairs in which His is neutral (His^0–Arg/Lys), the strengths of CH–π interactions approach those of cation–π interactions, on average (**Fig. 6B**). This is also evident for the reference cationic–aromatic pairs (Phe/Tyr/Trp–Arg/Lys). To elucidate whether the similarity in strength is a result of co-occurring contributions from CH–π and cation–π interactions or arises solely from one of these interaction types, we further inspected the interactions map of His–

Lys/Arg (**Fig. 5A–B**). We observed dual contributions from both types of interactions in His^0–Lys pairs (**Fig. 5A**), regardless of the geometric parameters (3 Å < D < 0 Å; 5° < θ_1 < 40°), however, these dual contributions are unpopulated (which we argue occurs because of a preference for H-bonding, see previous section). For the His–Arg case (**Fig. 5B**), we observe the occurrence of both standalone contributions (**Fig. 5**, geometry 15) and overlapping conformations (D ≈ 4.4; θ_1 ≈ 25°), with the latter being infrequently observed.

References

[1] Comparing Counterpoise-Corrected, Uncorrected, and Averaged Binding Energies for Benchmarking Noncovalent Interactions
Lori A. Burns, Michael S. Marshall, and C. David Sherrill
Journal of Chemical Theory and Computation 2014 10 (1), 49-57
DOI: 10.1021/ct400149j
[2] Emmanouil Semidalas, Golokesh Santra, Nisha Mehta, Jan M. L. Martin; S66 noncovalent interactions benchmark re-examined: Composite localized coupled cluster approaches. AIP Conference Proceedings 23 November 2022; 2611 (1): 020016. https://doi.org/10.1063/5.0119282
